# Identification of the Chemical Constituents of an Anti-Arthritic Chinese Medicine Wen Luo Yin by Liquid Chromatography Coupled with Mass Spectrometry

**DOI:** 10.3390/molecules24020233

**Published:** 2019-01-10

**Authors:** Huanyu Guan, Xiaomei Luo, Xiaoyan Chang, Meifeng Su, Zhuangzhuang Li, Pengfei Li, Xiaoming Wang, Yue Shi

**Affiliations:** 1 Institute of Medicinal Plant Development, Chinese Academy of Medical Sciences and Peking Union Medical College, Beijing 100193, China; guanhuanyu630@163.com (H.G.); Luoxiaomei1019@163.com (X.L.); changxiaoyan1234@163.com (X.C.); 20160931861@bucm.edu.cn (M.S.); lizz1213@126.com (Z.L.); lipengfei1121@126.com (P.L.); lmlwxm123@163.com (X.W.); 2 School of Pharmaceutical Sciences, Guizhou Medical University, Guiyang 550004, China

**Keywords:** Wen Luo Yin, chemical profiling, mass spectrometry, liquid chromatography

## Abstract

Wen Luo Yin (WLY), a well-known traditional Chinese medicine formulation, has been used as a complementary therapy for the treatment of rheumatoid arthritis in clinical settings. However, the chemical constituents of WLY remain unclear. In this study, a high-performance liquid chromatography coupled with tandem mass spectrometry method was established to separate and comprehensively identify the chemical constituents of WLY. The analytes were eluted with a mobile phase of acetonitrile and 0.1% aqueous acetic acid. Mass detection was performed in both positive and negative ion mode. The MS/MS fragmentation pathways were proposed for the identification of the components. A total of 42 compounds including sesquiterpenes, alkaloids, biflavonoids, polyacetylenes, phenylpropanoids and acetylenic phenols were identified unambiguously or tentatively according to their retention times and mass behavior with those of authentic standards or literature data. The identification and structural elucidation of chemical constituents may provide important information for quality control and pharmacological research of WLY.

## 1. Introduction

Rheumatoid arthritis (RA), a chronic inflammatory disease, is characterized by joint swelling, joint tenderness and destruction of synovial joints, resulting in severe disability and premature mortality [1]. Most of the symptoms of RA are distinguished as cold or hot according to the diagnostic guidelines of traditional Chinese medicine (TCM) [2]. Disease-modifying anti-rheumatic drugs (DMARDs) are the most commonly used treatments of RA [3]. However, because of the adverse effects and potential risks associated with DMARDs [4,5], the search for new anti-rheumatic drugs with high efficacy and low toxicity has attracted substantial attention. TCMs as complementary therapies have been demonstrated to be effective in the treatment of RA [6] and they are widely used in the modern clinic practice.

Wen Luo Yin (WLY) originally comes from the formulae of Guizhi Fuzi Tang, Zhu Fu Tang and Gancao Fuzi Tang as chronicled in “Jin Kui Yao Lve” written by Zhang Zhongjing (150–219 A.D.) [7]. WLY is given orally to patients as complementary therapy to treat RA in clinical settings. WLY is prepared using four herbs according to cold-hot theory of TCM, that is, processed *Radix Aconiti Lateralis Preparata* (“Fuzi” in Chinese, the lateral roots of *Aconitum Carmichaeli Debx*.), *Atractylodes macrocephala* koidz (“Baizhu” in Chinese, the rhizome of *A. macrocephala*), *Selaginella tamariscina* (Beauv) Spring (“Juanbai” in Chinese, the whole plant of *S. tamariscina*) and *Cinnamomi ramulus* (“Guizhi” in Chinese, the dried twig of *Cinnamomum cassia* Presl) in the ratio of 10:15:9:12 on a dry weight basis [8]. The ethanol extract of WLY, which acts on the cold symptoms of RA [9], has been reported to alleviate ankle pain and swelling and decrease the arthritis index of collagen-induced arthritis (CIA) in rats [10]. Moreover, the mechanism of the anti-arthritic action of WLY has been associated with reducing the expression of angiogenic activators [8]. Although the anti-arthritic activity of WLY is notable, only 7 of its chemical constituents—namely, 3 phenylpropanoids, 3 alkaloids and one biflavonoid—have been identified by comparing their retention times with those of reference standards using LC coupled with a photodiode array detector (DAD) [8]. The other chemical components of WLY are still unknown. The various constituents in WLY those are key to its efficacy still need to be explored by advanced analytical techniques. The main constituents of the four individual herbs used to prepare WLY have been reported to include alkaloids [11], flavonoids [12,13], sesquiterpenes [14,15], volatile oils and organic acids [16]. In our previous study, biflavonoids from *S. tamariscina* were characterized and identified by LC-DAD-ESI-MS [17]. However, an integrated investigation of the profile all the components of WLY has not been reported.

Therefore, the present study aimed to develop an HPLC-MS/MS method to comprehensively identify the chemical constituents in WLY. A total of 42 compounds including sesquiterpenes, alkaloids, biflavonoids, polyacetylenes, phenylpropanoids and acetylenic phenols were unambiguously identified or tentatively characterized.

## 2. Results and Discussion

### 2.1. Optimized Analytical Conditions

To improve resolution, decrease the run time and increase sensitivity, methanol, acetonitrile, ammonium acetate, formic acid and acetic acid were tested as potential mobile phases. Acetonitrile and 0.1% aqueous acetic acid were found to be the optimal mobile phase as they gave the best separation of the major constituents in WLY. Moreover, acetic acid improved the peak shapes and intensities for these components.

To obtain abundant mass information and high responses for most compounds in WLY, both positive and negative ion modes were employed to gain corresponding signals for structural characterization.

### 2.2. HPLC-MS/MS Analysis of the WLY Extract

**Figure 1:**
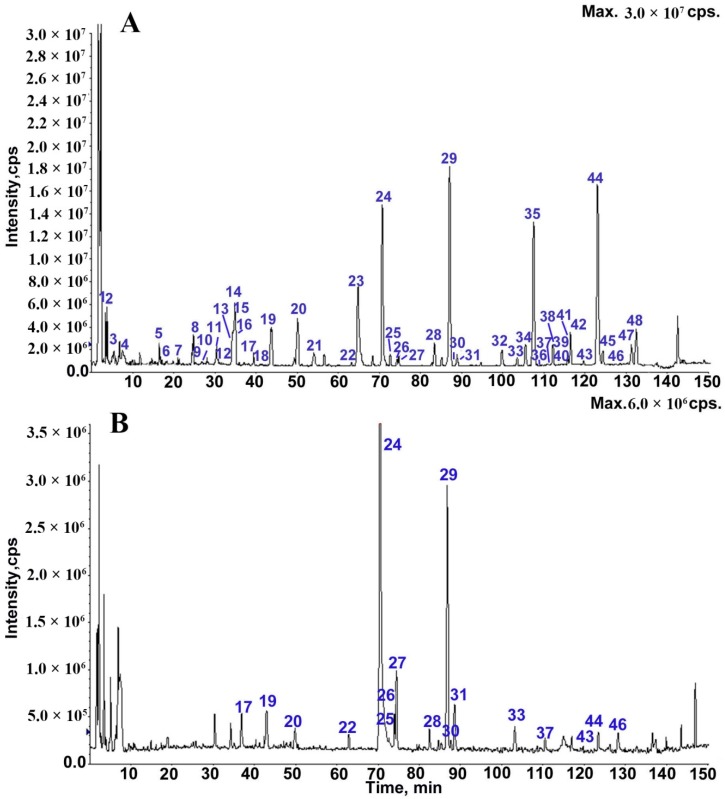
The TICs of WLY by LC-MS in the positive (**A**) and negative (**B**) modes.

The mass spectra of the reference standards were analyzed to characterize the typical constituents of WLY. The proposed fragmentation patterns of these reference standards were helpful for identifying constituents of WLY with similar fragmentation patterns. Although positive and negative ion modes were employed, higher sensitivities and clearer mass spectra were observed in the positive ion mode. The total ion current (TIC) chromatograms corresponding to positive and negative signals of WLY are shown in Figure 1. To avoid repetition, only the fragment ions in the positive mode are listed in Supplementary Table S1. A total of 48 peaks were detected by HPLC-MS/MS, 42 of which were identified. Compared to the compounds identified in the literature [8], 36 constituents are being reported in WLY by LC-MS/MS analysis for the first time. Selaginellin was tentatively characterized by MS/MS data for the first time.

**Figure 2:**
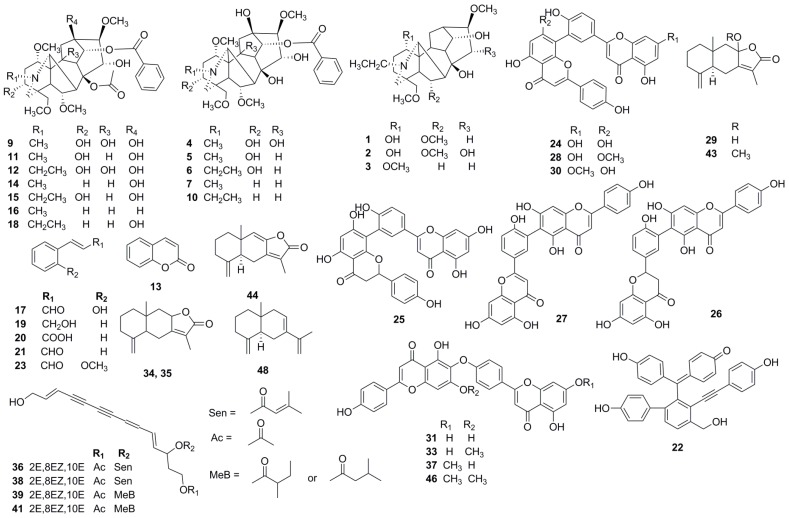
The chemical structures of compounds identified in WLY.

Thirteen peaks in the HPLC-MS/MS chromatograms were unambiguously identified by comparison of their retention times and MS data with those of authentic standards. The other 29 peaks were tentatively characterized by comparing their molecular weight and mass fragmentation pattern in their MS/MS spectra with published literature data. These identified constituents included sesquiterpenes, alkaloids, flavonoids, polyacetylenes, phenylpropanoids and acetylenic phenol compounds (Figure 2). The fragmentation pathways of the identified constituents are proposed in the Supplementary Figure S1.

#### 2.2.1. Identification of the Sesquiterpenes in WLY

**Figure 3:**
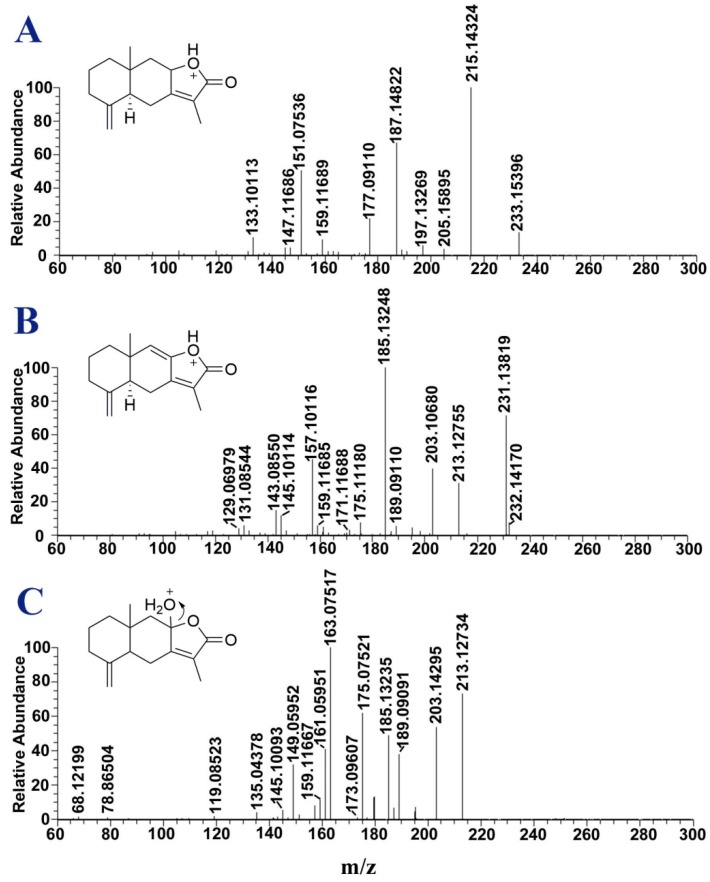
MS^n^ spectra of atractylenolide I (**A**); atractylenolide II (**B**) and atractylenolide III (**C**).

The MS/MS data of the 3 sesquiterpenes (Figure 3) were acquired using a high-resolution linear ion trap Orbitrap mass spectrometer (Thermo Fisher, Waltham, MA, USA). In the positive MS^n^ spectra, atractylenolides I, II and III displayed [M + H]^+^ ions at *m*/*z* 233.1540, 231.1382 and 249.1482, respectively. The fragment ions of the corresponding sesquiterpenes were [M + H − H_2_O]^+^ and [M + H − H_2_O − CO]^+^, which are characteristic of sesquiterpenes containing γ-lactone moieties. Notably, the loss of a C_2_H_4_ fragment (28.0313 Da) was due to the characteristic fragmentation pathway of these sesquiterpenes involving the rearrangement of the twelve-membered-ring, which could be distinguished from the loss of a CO fragment (27.9949 Da). The fragment ions at *m*/*z* 177.0911 of atractylenolide I, 175.1118 of atractylenolide II and 175.0752 of atractylenolide III were attributed to [M + H − C_4_H_8_]^+^, [M + H – CO − C_2_H_4_]^+^ and [M + H − H_2_O − C_4_H_8_]^+^, respectively. Moreover, a McLafferty rearrangement resulted in the loss of a C_3_H_6_ fragment after cleavage of the A-ring through a four-membered-ring rearrangement.

Based on the fragmentation patterns, the chemical structures of compounds corresponding to peaks 29, 34, 35, 43, 44 and 48 in the complex mixtures were identified or tentatively characterized. Peaks 29, 35 and 44 were attributed to atractylenolide III, I and II, respectively, by comparing their retention times (t_R_) and MS^n^ spectra with those of the reference standards. Peak 34 (t_R_ = 105.5 min) at *m*/*z* 233 showed the same fragments as those of atractylenolide I and was identified as its geometric isomer, isoatractylenolide I [18]. Peak 43 exhibited an [M + H]^+^ ion at *m*/*z* 263, which was 14 Da more massive than that of atractylenolide III. The product ions of peak 43 at *m*/*z* 231 ([M + H − CH_3_OH]^+^), 213 ([M + H − CH_3_OH − H_2_O]^+^), 203 ([M + H − CH_3_OH − CO]^+^), 185 ([M + H − CH_3_OH − CO − H_2_O]^+^) and 163 ([M + H − CH_3_OH − C_5_H_8_]^+^) were same as those of atractylenolide III. Therefore, peak 43 was attributed to 8-methoxyatractylenolide I [19]. Peak 48 eluted at 132.8 min and displayed an [M + H]^+^ ion at *m*/*z* 203. The fragment ion of peak 48 at *m*/*z* 161 was formed by the loss of C_3_H_6_ from the precursor ion via a McLafferty rearrangement. The fragment ion at *m*/*z* 161 then lost a C_2_H_4_ fragment to produce an ion at *m*/*z* 133. By comparing these data with the literature data [15], peak 48 was attributed to atractylenolide VI.

#### 2.2.2. Identification of the Alkaloids in WLY

A total of 15 aconitum alkaloids were unambiguously or tentatively identified and were classified into diester diterpenoid aconitines (DDAs), monoester diterpenoid aconitines (MDAs) and non-ester alkaloids (NEAs). For the DDAs, aconitine and hypaconitine standards were used to characterize the fragments pathways in the MS^n^ spectra of aconitum alkaloids. In the positive mode, the molecular ions produced the characteristic ions via the successive or simultaneous loss of the one or more CH_3_OH (32 Da), CH_3_COOH (60 Da), CO (28 Da) or C_6_H_5_COOH (122 Da) fragments. For example, the product ion of hypaconitine at *m*/*z* 556 was generated from the molecular ion at *m*/*z* 616 by the loss of a CH_3_COOH fragment, which subsequently lost a CH_3_OH group to form the product ion at *m*/*z* 524. The product ion at *m*/*z* 496 was formed by the loss of a CO fragment from the product ion at *m*/*z* 524. The product ion at m/z 338 was attributed to [M + H − CH_3_COOH − 3CH_3_OH − C_6_H_5_COOH]^+^. Peaks 14 (t_R_ = 34.5 min) and 15 (t_R_ = 35.3 min) were unambiguously attributed to hypaconitine and aconitine by comparison of their experimental data to those of the reference standards. Similar fragmentation pathways were observed in the spectra of peaks 9, 11, 12, 16 and 18. Peak 11 displayed a molecular ion at *m*/*z* 632, which was 16 Da more massive than that of hypaconitine. The product ions at *m*/*z* 572 ([M + H − CH_3_COOH]^+^), 540 ([M + H − CH_3_COOH − CH_3_OH]^+^) and 354 [M + H − CH_3_COOH − 3CH_3_OH − BzOH]^+^ were also 16 Da higher than the corresponding fragment ions of hypaconitine, indicating that a hydroxyl group was present in the compound corresponding to peak 11. By comparison with the reported data [20], peak 11 (t_R_ = 30.6 min) was tentatively attributed to mesaconitine. The molecular ions and characteristic product ions of peaks 16 (t_R_ = 35.4 min) and 18 (t_R_ = 40.1 min) were 16 Da lower than the corresponding ions of hypaconitine and aconitine. Based on previously reported data, peaks 16 and 18 were tentatively attributed to 13-deoxyhypaconitine [21] and deoxyaconitine [20], respectively. Peaks 9 (t_R_ = 25.2 min) and 12 (t_R_ = 31.1 min) were tentatively attributed to 10-OH-mesaconitine [11,20] and 10-OH-aconitine [20] by comparing their MS^n^ spectra with those of peak 11 and aconitine, respectively.

Structurally, MDAs are diterpenoid alkaloids with a hydroxyl group at the C-8 position. Compared with the fragmentations of DDAs, the losses of a H_2_O unit and one or more CH_3_OH fragments are commonly observed in the fragmentation patterns of MDAs. In the positive mode, peaks 4 (t_R_ = 8.8 min), 5 (t_R_ = 16.2 min), 6 (t_R_ = 18.7 min), 7 (t_R_ = 21.4 min) and 10 (t_R_ = 25.2 min) showed protonated molecular ions at *m*/*z* 606, 590, 604, 574 and 588, respectively. The protonated molecular ion of peak 4 gave four fragment ions at *m*/*z* 588, 574, 556 and 524, which were assigned as [M + H − H_2_O]^+^, [M + H − CH_3_OH]^+^, [M + H − H_2_O − CH_3_OH]^+^ and [M + H − H_2_O − 2CH_3_OH]^+^, respectively. Based on comparisons to the available data [22], peak 4 was tentatively characterized as 14-benzoxyl-10-OH-mesaconine. Similar fragmentation patterns were observed for peaks 5, 6, 7 and 10. Based on the data reported in the literatures [21,22,23,24,25], peaks 5, 6, 7 and 10 were tentatively identified as 14-benzoylmesaconine, 14-benzoylaconine, 14-benzoylhypaconine and 14-benzoyldeoxyaconine, respectively.

The NEAs possessed molecular weights less than 500 Da and eluted within the first 5 min. Peaks 1 (t_R_ = 2.4 min) and 2 (t_R_ = 3.6 min) showed protonated molecular ions at *m*/*z* 438 and 454. The most prominent fragment ions at *m*/*z* 420 of peak 1 and *m*/*z* 436 of peak 2 as the diagnostic ions [25] were produced through dehydration of the protonated molecular ions at C-1, implying that peaks 1 and 2 were C-1-hydroxyl-substituted NEAs. By comparing the protonated molecular ions and fragments ions with those reported in the literature [26], peaks 1 and 2 were tentatively identified as neoline and fuziline. Peak 3 eluted at 4.6 min and exhibited a protonated molecular ion at *m*/*z* 422. The base fragment ion of peak 3 at *m*/*z* 390 was assigned as [M + H − CH_3_OH]^+^. Other fragment ions of peak 3 at *m*/*z* 372 ([M + H − CH_3_OH − H_2_O]^+^) and 358 ([M + H − 2CH_3_OH]^+^) were observed. Based on a comparison to the literature data [24], peak 3 was tentatively attributed to talatizamine.

#### 2.2.3. Identification of the Biflavonoids in WLY

Biflavonoids are a class of compounds consisting two flavonoid moieties connected via a C–O–C or a C–C bond. The biflavonoids of WLY eluted at approximately 70–129 min and exhibited protonated molecular ions from 500–600 Da. The fragment pathways of biflavonoids were characterized in our previous study [17] and the characteristic fragment ions were generated by losses of H_2_O, CO, CO_2_, C_2_H_2_O and C_3_O_2_ fragments. Retro-Diels-Alder (RDA) reactions were also observed in the fragmentation pathways of biflavonoids.

The characteristic fragmentation pathway of amentoflavone-type biflavonoids, including the cleavage of the C-ring from the second flavone moiety at the 0/4 position, was observed in peaks 24, 25, 28 and 30. Peaks 24 (t_R_ = 70.1 min) and 30 (t_R_ = 88.0 min) were unambiguously identified as amentoflavone and 7-*O*-methylamentoflavone by comparing their experimental data with those of reference standards. Peak 25 eluted at 70.8 min and possessed a protonated molecular ion at *m*/*z* 541 ([M + H]^+^), which was 2 Da more massive than that of amentoflavone, implying that two additional hydrogen atoms were present. The series of prominent fragment ions of peak 25 at *m*/*z* 421 (^1,3^IIA^+^), 403 (^1,3^IIA^+^ − H_2_O), 337 (^1,3^IIA^+^ − 2C_2_H_2_O), 311 (^1,3^IIA^+^ − C_2_H_2_O − C_3_O_2_) and 283 (^1,3^IIA^+^ − C_2_H_2_O − C_3_O_2_ − CO) indicated the instability of the C-ring at the 1/3 position of the second flavone moiety in the mass experiment as a result of the reduction of the double bond [27]. The two additional hydrogen atoms were deduced to be located at C-2″ and C-3″. Peak 25 was tentatively attributed to 2″,3″-dihydroamentoflavone. Peak 28 (t_R_ = 82.9 min) showed a protonated molecular ion at *m*/*z* 553, which is the same mass as that of 7-*O*-methylamentoflavone. The observed fragment ion of peak 28 at *m*/*z* 153 (^1,3^IA^+^) was 14 Da lower than that of the corresponding ion of 7-*O*-methylamentoflavone, implying a methoxy group was located on the A-ring of the second flavonoid moiety. Peak 28 was tentatively identified as 7″-*O*-methylamentoflavone.

Compared to the fragmentation pathways of amentoflavone-type biflavonoids, RDA fragmentation of the first flavone moiety of robustaflavone-type biflavonoids were more common. Peak 27 (t_R_ = 74.2 min), possessing a protonated molecular ion at *m*/*z* 539, was unambiguously identified as robustaflavone by comparing its experimental data with those of the reference standard. Peak 26 eluted at 73.6 min and exhibited a protonated molecular ion at *m*/*z* 541, which was 2 Da more massive than that of robustaflavone. Moreover, the fragment ions at *m*/*z* 415 (^1,4^IB^+^) and 389 (^1,3^IB^+^) were 2 Da more massive than the corresponding ions of robustaflavone. The data implied that additional hydrogen atoms are located in the C-ring of the first flavone moiety. Peak 26 was tentatively attributed to 2,3-dihydrorobustaflavone.

Hinokiflavone-type biflavonoids comprise two flavonoid moieties connected by a C–O–C bond. The characteristic odd-electron fragment ions of hinokiflavone-type biflavonoids were produced by the cleavage of the bridging C–O bond. For example, peak 31 eluted at 89.0 min and exhibited a protonated molecular ion at *m*/*z* 539. A series of odd-electron fragment ions at 286 ([Flavone II + OH]^+•^), 270 ([Flavone II]^+•^ or [Flavone I]^+•^) and 254 ([Flavone I − O]^+•^) were observed in the MS^n^ spectrum of peak 31. Peak 31 was unambiguously identified as hinokiflavone by comparison of its experimental data with those of the reference standard. Similarly, a series of odd-electron fragment ions at *m*/*z* 284 ([Flavone II]^+•^) and 254 ([Flavone I − O]^+•^) of peak 33 (t_R_ = 103.5 min); 286 ([Flavone II + OH]^+•^), 270 ([Flavone II]^+•^) and 268 ([Flavone I − O]^+•^) of peak 37 (t_R_ = 111.4 min); and 284 ([Flavone II]^+•^ or [Flavone I]^+•^), 268 ([Flavone I − O]^+•^) and 256 ([Flavone II or I − CO]^+•^) of peak 46 (t_R_ = 128.1 min) were also observed. Based on comparisons with the reported data [17], peaks 33, 37 and 46 were tentatively attributed to isocryptomerin, neocryptomerin and 7,7″-di-*O*-methylhinokiflavone, respectively.

#### 2.2.4. Identification of the Polyacetylenes in WLY

**Figure 4:**
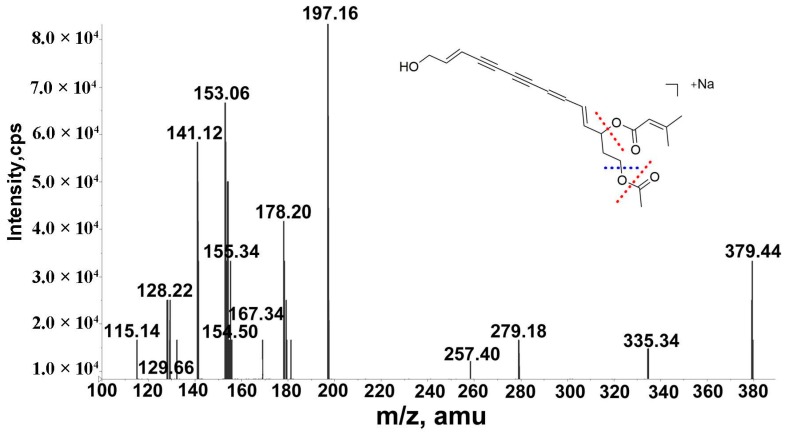
MS^n^ spectrum of 14-acetoxy-12-senecioyloxytetradeca-2E,8EZ,10E-trien-4,6-diyn-1-ol.

A total of 4 polyacetylenes were tentatively identified from WLY. In the MS^n^ spectra, the product ions were commonly generated by the loss of the acyl side chains at C-12. The [M + Na]^+^ ion of peak 36, which eluted at 107.8 min, was found at *m*/*z* 379. The fragment ions at *m*/*z* 279 and 257 were formed by loss of a senecioic acid unit (SenOH, 100 Da) and a SenONa unit (122 Da), respectively. The product ion at *m*/*z* 257 further lost a unit of acetic acid to generate the ion at *m*/*z* 197. Peak 38 eluted at 112.2 min and afforded the same [M + Na]^+^ ion and fragment ions as those of peak 36. Peaks 36 and 38 were tentatively attributed to a pair of the geometric isomers of 14-acetoxy-12-senecioyloxytetradeca-2E,8EZ,10E-trien-4,6-diyn-1-ol [14]. The MS^n^ data of the above compound are shown in Figure 4. A similar fragmentation pathway was observed for peaks 39 (t_R_ = 113.0 min) and 41 (t_R_ = 115.6 min). Peaks 39 and 41 showed the same [M + Na]^+^ ion at *m*/*z* 381 and the same product ions. By comparisons with the MS data of peaks 36 and 38 and literature data, peaks 39 and 41 were tentatively attributed to a pair of geometric isomers of 14-acetoxy-12-methylbutyryl-tetradeca-2E,8EZ,10E-trien-4,6-diyn-1-ol [28].

#### 2.2.5. Identification of the Phenylpropanoids in WLY

The phenylpropanoids in WLY eluted between 33.0 and 64.2 min and exhibited protonated molecular ions less than 200 Da. Peaks 13, 19, 20 and 21 eluted at 33.2, 43.5, 49.3 and 53.8 min were unambiguously identified as coumarin, cinnamic alcohol, cinnamic acid and cinnamic aldehyde, respectively, by comparison of their experimental data with those of reference standards. Based on their similar fragmentation patterns and the reported data [29,30], the compounds corresponding to peaks 17 (t_R_ = 36.7 min) and 23 (t_R_ = 64.2 min) were tentatively identified as 2-hydroxycinnamaldehyde and 2-methoxycinnamaldehyde. Specifically, the characteristic fragment ions were formed by losses of H_2_O (18 Da) and CO (28 Da). A series of fragment ions at *m*/*z* 91 ([C_7_H_7_]^+^), 77 ([C_6_H_5_]^+^) and 65 ([C_5_H_5_]^+^) were observed. For example, peak 17 exhibited an [M + H]^+^ ion at *m*/*z* 149. The fragment ions at *m*/*z* 131, 121, 103, 93, 91 and 77 were assigned to [M + H − H_2_O]^+^, [M + H − CO]^+^, [M + H − CO − H_2_O]^+^, [M + H − CO − C_2_H_4_]^+^, [C_7_H_7_]^+^ and [C_6_H_5_]^+^, respectively.

#### 2.2.6. Identification of the Acetylenic Phenol Compound in WLY

**Figure 5:**
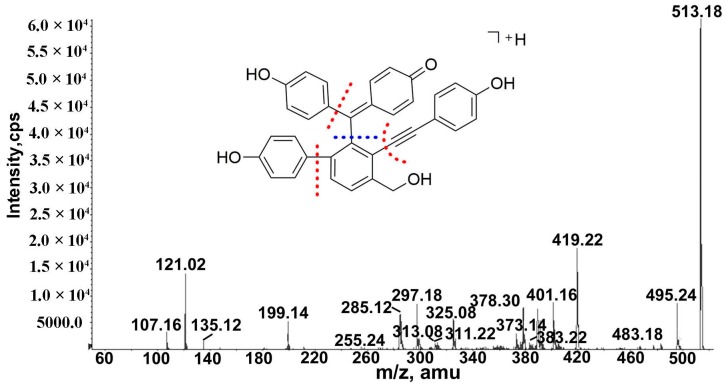
MS^n^ spectrum of selaginellin.

Selaginellins, characteristic compounds of the genus *Selaginella*, consist of five six-membered unsaturated rings and an acetylenic bridge. Peak 22 (t_R_ = 62.6 min) exhibited an [M + H]^+^ ion at *m*/*z* 513, which yielded fragments ions at *m*/*z* 419 and 495 by the losses of a C_6_H_5_OH fragment (94 Da) and H_2_O (18 Da), respectively. The fragment ion at *m*/*z* 419 went on to lose a C_6_H_5_OH fragment and H_2_O to generate the ions at *m*/*z* 325 and 401, respectively. Moreover, the ion at *m*/*z* 297 and the odd-electron fragment ions at *m*/*z* 378 were formed by losses of C_13_H_10_O_2_ (198 Da) and C_8_H_5_O∙(117 Da) fragments from the ion at *m*/*z* 495. Based on a comparison to the literature data [31], peak 22 was tentatively attributed to selaginellin. The MS/MS fragmentation data (Figure 5) and pathway of selaginellin was proposed for the first time.

## 3. Material and Methods

### 3.1. Chemicals and Reagents

Processed *Radix Aconiti Lateralis Preparata* (lot number 080306), *A. macrocephala* (lot number 090326), *S. tamariscina* (lot number 60300511) and *C. Ramulus* (lot number 090328) were purchased from Beijing Tongren Tang Pharmaceutical Co. Ltd. (Beijing, China). These samples were identified by Prof. Sisong Yi as the lateral roots of *Aconitum Carmichaeli Debx*., the rhizome of *A. macrocephala*, the whole plant of *S. tamariscina* and the twig of *Cinnamomum cassia* Presl. The reference standards of aconitine and hypaconitine were purchased from the National Institute for the Control of Pharmaceutical and Biological Products (Beijing, China). Cinnamic aldehyde, cinnamic acid, cinnamic alcohol, coumarin and atractylenolides I, II and III were purchased from Sichuan Weikeqi Biological Technology Co. Ltd. (Chengdu, Sichuan, China). Amentoflavone was purchased from Sichuan Xiya Chemical Co., Ltd. (Chengdu, Sichuan, China). Robustaflavone was supplied by Prof. Keli Chen from Hubei University of Chinese Medicine. Hinokiflavone was provided by Prof. Weisheng Feng from Henan University of Chinese Medicine. 7-*O*-methylamentoflavone was provided by Prof. Lijuan Chen from Sichuan University. The purities of the reference standards were determined to be no less than 98% by HPLC-DAD.

HPLC-grade acetonitrile were purchased from Honeywell Burdick & Jackson Company (Morristown, NJ, USA). Acetic acid (HPLC-grade) was obtained from TEDIA Company Inc. (Fairfield, OH, USA). Deionized water for HPLC analysis was prepared using a Milli-Q water purification system (Milford, MA, USA). All other reagents were of analytical grade.

### 3.2. Sample Preparation

The medicinal herbs, processed *Radix Aconiti Lateralis Preparata*, *A. macrocephala*, *S. tamariscina* and *C. Ramulus*, were mixed according to the previously described formula and pulverized into a powder. The obtained powder was dispersed in a volume of 75% ethanol ten times greater than the weight of powder for 30 min and then the mixture was refluxed twice for 1 h each time. After filtration, the filtrates were combined and concentrated to afford the extract. A sample of the WLY extract (10 mg) was dissolved in 1 mL of 60% methanol. After centrifugation at 10,033× *g* for 10 min, a 20 µL aliquot of the solution was injected into the HPLC-MS/MS system. Three individual samples were analyzed using the proposed LC-MS/MS conditions.

### 3.3. Analytical Method

An Agilent 1100 HPLC system (Agilent, MA, USA) consisting of a vacuum degasser, a quaternary pump and an auto-sampler coupled to an API 3200 QTRAP mass spectrometer (Applied Biosystems/MDS SCIEX, Concord, ON, Canada) was used for qualitative analysis. Chromatographic separation was achieved on a Waters C18 analytical column (4.6 mm × 250 mm, 5 μm, Waters, Milford, MA, USA) using a gradient elution system with a mobile phase of acetonitrile (A) and 0.1% aqueous acetic acid (B) at a flow rate of 1 mL/min. The gradient elution program was as follows: 10–30% A from 0–55 min, 30–40% A from 55–75 min, 40–50% A from 75–105 min, 50–60% A from 105–130 min, 60–90% A from 130–140 min, 90% A from 140–150 min. The column temperature was 30 °C and the injection volume was 20 μL.

The MS conditions were as follows: data were collected in the positive and negative ion modes from *m*/*z* 100 to 1000 amu; curtain gas (CUR): 15.0 psi; collision gas (CAD): medium; ion spray voltage (IS): −4500 V (in negative ionization mode) and 4500 V (in positive ionization mode); source temperature: 450 °C; nebulizer gas (GS1): 70 psi; auxiliary gas (GS2): 60 psi; declustering potential (DP): 55 V; collision energy (CE): 32 eV; entrance potential (EP): 10 V; and collision cell exit potential (CXP): 3 V.

### 3.4. Data Analysis

Data analysis for the identification of constituents was performed using the AB SCIEX Analyst 1.5 Software (AB SCIEX, Redwood City, CA, USA).

## 4. Conclusions

Because of their low concentrations and similar structures, the identification of the major constituents of WLY formulae by traditional phytochemical and analytical methods is challenging. In this study, a sensitive and accurate HPLC-MS/MS method was established to comprehensively and systematically identify the chemical constituents of WLY. A total of 42 compounds, namely, 6 sesquiterpenes, 15 alkaloids, 10 biflavonoids, 4 polyacetylenes, 6 phenylpropanoids and 1 acetylenic phenol, were identified by comparing their retention times and mass data with those of reference standards and literature data. The fragmentation patterns of the 42 identified constituents of WLY were proposed. The MS/MS data of selaginellin and the fragmentation pathways of polyacetylenes and selaginellin are reported for the first time. Moreover, detailed analyses of the fragmentation pathways of atractylenolides I, II and III were carried out. These qualitative results provide essential data for the quality control and the metabolites identification of WLY. These results will also be useful for the analysis of formulae analogous to WLY.

## Supplementary Materials

The following are available online in the supporting information tab for this article. Figure S1: The proposed fragmentation pathways of the identified compounds from WLY. Table S1: Characterization of the constituents of WLY by LC-MS/MS.
