# Supplementary material for: Identification of the Chemical Constituents of an Anti-Arthritic Chinese Medicine Wen Luo Yin by Liquid Chromatography Coupled with Mass Spectrometry
Source: Molecules. 2019 Jan 10;24(2):233. doi: 10.3390/molecules24020233 (PMC6359360; doi:10.3390/molecules24020233)
Supplement: Supplementary file 1 [file molecules-24-00233-s001.pdf]

Supporting Materials

Identification of the Chemical Constituents of an Anti-Arthritic Chinese Medicine Wen Luo Yin by Liquid Chromatography Coupled with Mass Spectrometry

Huanyu Guan [1,2], Xiaomei Luo [1], Xiaoyan Chang [1], Meifeng Su [1], Zhuangzhuang Li [1], Pengfei Li [1], Xiaoming Wang [1] and Yue Shi [1,*]

[1] Institute of Medicinal Plant Development, Chinese Academy of Medical Sciences and Peking Union Medical College, Beijing 100193, China; guanhuanyu630@163.com (H.G.); Luoxiaomei1019@163.com (X.L.); changxiaoyan1234@163.com (X.C.); 20160931861@bucm.edu.cn (M.S.); lizz1213@126.com (Z.L.); lipengfei1121@126.com (P.L.); lmlwxm123@163.com (X.W.)

[2] School of Pharmaceutical Sciences, Guizhou Medical University, Guiyang 550004, China

* Correspondence: shiyue1029@126.com; Fax: +86-(0)10-5783-3270

Received: 09 December 2018; Accepted: 08 January 2019; Published: 10 January 2019

Table 1. Characterization of the constituents of WLY by LC-MS/MS.

Peak no.	t_R (min)	Identification	Formula	$[M+H]^+$ (m/z)	$[M-H]^-$ (m/z)	Fragment ions in positive ion mode	Structure class	Plant material
1	2.4	neoline	$C_{24}H_{39}NO_6$	438.4	—	420$[M+H-H_2O]^+$,388$[M+H-H_2O-CH_3OH]^+$	alkaloid	RA
2	3.6	fuziline	$C_{24}H_{39}NO_7$	454.4	—	436$[M+H-H_2O]^+$,418$[M+H-2H_2O]^+$,404$[M+H-H_2O-CH_3OH]^+$, 386$[M+H-2H_2O-CH_3OH]^+$,372$[M+H-H_2O-2CH_3OH]^+$	alkaloid	RA
3	4.6	talatizamine	$C_{24}H_{39}NO_5$	422.6	—	390$[M+H-CH_3OH]^+$,372$[M+H-CH_3OH-H_2O]^+$, 358 ($[M+H-2CH_3OH]^+$)	alkaloid	RA
4	8.8	14-benzoyl-10-OH-mesaconine	$C_{31}H_{43}NO_{11}$	606.4	—	588($[M+H-H_2O]^+$),574($[M+H-CH_3OH]^+$), 556($[M+H-H_2O-CH_3OH]^+$),524($[M+H-H_2O-2CH_3OH]^+$)	alkaloid	RA
5	16.2	benzoylmesaconine	$C_{31}H_{43}NO_{10}$	590.6	—	572($[M+H-H_2O]^+$), 558($[M+H-CH_3OH]^+$), 540($[M+H-H_2O-CH_3OH]^+$),508($[M+H-H_2O-2CH_3OH]^+$)	alkaloid	RA
6	18.7	14-benzoylaconine	$C_{32}H_{45}NO_{10}$	604.4	—	586($[M+H-H_2O]^+$),572($[M+H-CH_3OH]^+$), 554($[M+H-H_2O-CH_3OH]^+$),522($[M+H-H_2O-2CH_3OH]^+$)	alkaloid	RA
7	21.4	14-benzoylhypaconine	$C_{31}H_{43}NO_9$	574.6	—	542($[M+H-CH_3OH]^+$), 510($[M+H-2CH_3OH]^+$),492($[M+H-H_2O-2CH_3OH]^+$)	alkaloid	RA
8	24	unknown		537.6	—	417,375		RA
9	25.2	10-OH-mesaconitine	$C_{33}H_{45}NO_{12}$	648.4	—	588($[M+H-CH_3COOH]^+$),556($[M+H-CH_3COOH-CH_3OH]^+$), 528($[M+H-CH_3COOH-CH_3OH-CO]^+$)	alkaloid	RA
10	25.2	14-benzoyldeoxyaconine	$C_{32}H_{45}NO_9$	588.2	—	556($[M+H-CH_3OH]^+$), 538($[M+H-H_2O-CH_3OH]^+$), 524($[M+H-2CH_3OH]^+$), 506($[M+H-H_2O-2CH_3OH]^+$)	alkaloid	RA
11	30.6	mesaconitine	$C_{33}H_{45}NO_{11}$	632.6	—	572($[M+H-CH_3COOH]^+$),540($[M+H-CH_3COOH-CH_3OH]^+$), 354($[M+H-CH_3COOH-3CH_3OH-C_6H_5COOH]^+$)	alkaloid	RA

12	31.1	10-OH-aconitine	$C_{34}H_{47}NO_{12}$	662.4	—	602([M+H-CH_3COOH]$^+$),570([M+H-CH_3COOH-CH_3OH]$^+$), 384([M+H-CH_3COOH-3CH_3OH-C_6H_5COOH]$^+$)	alkaloid	RA
13	33.2	coumarin*	$C_9H_6O_2$	147.4	—	103([M+H-CO_2]$^+$),91([C_7H_7]$^+$),77([C_6H_5]$^+$),65([C_5H_5]$^+$)	phenylpropanoid	CR
14	34.5	hypaconitine*	$C_{33}H_{45}NO_{10}$	616.6	—	556([M+H-CH_3COOH]$^+$),524([M+H-CH_3COOH-CH_3OH]$^+$), 496([M+H-CH_3COOH-CH_3OH-CO]$^+$), 338([M+H-CH_3COOH-3CH_3OH-C_6H_5COOH]$^+$)	alkaloid	RA
15	35.3	aconitine*	$C_{34}H_{47}NO_{11}$	646.4	—	586([M+H-CH_3COOH]$^+$),554([M+H-CH_3COOH-CH_3OH]$^+$), 368([M+H-CH_3COOH-3CH_3OH-C_6H_5COOH]$^+$)	alkaloid	RA
16	35.4	13-deoxyhypaconitine	$C_{33}H_{45}NO_9$	600.2	—	540([M+H-CH_3COOH]$^+$),508([M+H-CH_3COOH-CH_3OH]$^+$), 322([M+H-CH_3COOH-3CH_3OH-C_6H_5COOH]$^+$)	alkaloid	RA
17	36.7	2-hydroxy cinnamaldehyde	$C_9H_8O_2$	149.4	147.2	131([M+H-H_2O]$^+$),121([M+H-CO]$^+$), 103 ([M+H-H_2O-CO]$^+$), 93([M+H-CO-C_2H_4]$^+$), 91([C_7H_7]$^+$), 77([C_6H_5]$^+$)	phenylpropanoid	CR
18	40.1	deoxyaconitine	$C_{34}H_{47}NO_{10}$	630.4	—	570([M+H-CH_3COOH]$^+$),538([M+H- CH_3COOH-CH_3OH]$^+$), 510([M+H-CH_3COOH-CH_3OH-CO]$^+$)	alkaloid	RA
19	43.5	cinnamic alcohol*	$C_9H_{10}O$	135.4	133.4	117 ([M+H-H_2O]$^+$), 91([C_7H_7]$^+$),	phenylpropanoid	CR
20	49.3	cinnamic acid*	$C_9H_8O_2$	149.4	147.2	131([M+H-H_2O]$^+$),103([M+H-H_2O-CO]$^+$),91([C_7H_7]$^+$),77([C_6H_5]$^+$)	phenylpropanoid	CR
21	53.8	cinnamic aldehyde*	C_9H_8O	133.4	—	115([M+H-H_2O]$^+$),105([M+H-CO]$^+$),91([C_7H_7]$^+$),77([C_6H_5]$^+$)	phenylpropanoid	CR
22	62.6	selaginellin	$C_{34}H_{24}O_5$	513.4	511.4	495([M+H-H_2O]$^+$),419([M+H-PhOH]$^+$),401([M+H-PhOH-H_2O]$^+$), 378([M+H-H_2O-C_8H_5O•]$^{+•}$),325([M+H-2PhOH]$^+$),297([M+H-H_2O-$C_{13}H_{10}O_2$]$^+$)	acetylenic phenol compound	ST
23	64.2	2-methoxy cinnamaldehyde	$C_{10}H_{10}O_2$	163.4	—	145([M+H-H_2O]$^+$),135([M+H-CO]$^+$), 115 ([M+H-H_2O-CH_2O]$^+$),107([M+H-CO-C_2H_4]$^+$)	phenylpropanoid	CR

24	70.1	amentoflavone*	$C_{30}H_{18}O_{10}$	539.4	537.2	497([M+H-C_2H_2O]$^+$),403(^{1,3} IIA$^+$-H_2O),377(^{0,4} IIA$^+$), 347 (^{1,3} IIA$^+$-H_2O-2CO), 335(^{0,4}IIA $^+$-C_2H_2O)	biflavonoid	ST
25	70.8	2”,3”-dihydroamentoflavone	$C_{30}H_{20}O_{10}$	541.2	539.2	421(^{1,3} IIA$^+$),403(^{1,3} IIA$^+$-H_2O), 337(^{1,3} IIA$^+$-2C_2H_2O), 311(^{1,3}IIA $^+$-C_2H_2O-C_3O_2),283(^{1,3}IIA $^+$-C_2H_2O-C_3O_2-CO)	biflavonoid	ST
26	73.6	2,3-dihydrorobustaflavone	$C_{30}H_{20}O_{10}$	541.4	539.2	415 (^{1,4}IB$^+$), 389 (^{1,3}IB$^+$), 153(^{1,3}IA$^+$),121(^{0,2}IIB$^+$)	biflavonoid	ST
27	74.2	robustaflavone*	$C_{30}H_{18}O_{10}$	539.4	537.2	521([M+H-H_2O]$^+$), 465([M+H-H_2O-2CO]$^+$), 413(^{1,4}IB $^+$), 403(^{1,3}IIA$^+$-H_2O), 387(^{1,3}IB $^+$), 153(^{1,3}IA$^+$),121(^{0,2}IIB$^+$)	biflavonoid	ST
28	82.9	7”-*O*-methylamentoflavone	$C_{31}H_{20}O_{10}$	553.4	551.2	521([M+H-CH_3OH]$^+$),417(^{1,3} IIA$^+$-H_2O), 401(^{1,3}IB $^+$), 391(^{0,4} IIA$^+$), 361(^{1,3} IIA$^+$-H_2O-2CO),153(^{1,3}IA$^+$), 121(^{0,2}IIB$^+$)	biflavonoid	ST
29	87.2	atractylenolide III*	$C_{15}H_{20}O_3$	249.1	247.2	231([M+H-H_2O]$^+$), 213([M+H-2H_2O]$^+$), 203([M+H-H_2O-CO]$^+$), 189([M+H-H_2O-C_3H_6]$^+$),185([M+H-2H_2O-CO]$^+$),175([M+H-H_2O-C_4H_6]$^+$),163([M+H-H_2O-C_5H_8]$^+$)	sesquiterpene	AM
30	88.0	7-*O*-methylamentoflavone*	$C_{31}H_{20}O_{10}$	553.4	551.2	417(^{1,3} IIA$^+$-H_2O), 391(^{0,4} IIA$^+$),361 (^{1,3} IIA$^+$-H_2O-2CO), 349(^{0,4}IIA $^+$-C_2H_2O), 167(^{1,3}IA$^+$), 121(^{0,2}IIB$^+$)	biflavonoid	ST
31	89.0	hinokiflavone*	$C_{30}H_{18}O_{10}$	539.2	537.2	387 (^{1,3}IB $^+$),286 ([Flavone II + OH]$^{+\bullet}$), 270 ([Flavone I] $^{+\bullet}$ or [Flavone II] $^{+\bullet}$), 257 ([Flavone II+O-CO]$^+$), 254 ([Flavone I-O]$^{+\bullet}$)	biflavonoid	ST
32	98.0	unknown		437.2	—	303		AM
33	103.5	isocryptomerin	$C_{31}H_{20}O_{10}$	553.4	551.2	521([M+H-CH_3OH]$^+$), 401(^{1,3}IB $^+$), 299 ([Flavone II+O]$^+$), 284 ([Flavone II]$^{+\bullet}$), 271 ([Flavone II+O-CO]$^+$), 254 ([Flavone I-O]$^{+\bullet}$)	biflavonoid	ST
34	105.5	isoatractylenolide I	$C_{15}H_{20}O_2$	233.4	—	215([M+H-H_2O]$^+$),187([M+H-H_2O-CO]$^+$),177([M+H-C_4H_8]$^+$), 159([M+H-CO-H_2O-C_2H_4]$^+$), 151([M+H-C_4H_8-C_2H_2]$^+$)	sesquiterpene	AM
35	107.6	atractylenolide I*	$C_{15}H_{20}O_2$	233.4	—	215([M+H-H_2O]$^+$),187([M+H-H_2O-CO]$^+$),177([M+H-C_4H_8]$^+$), 159([M+H-CO-H_2O-C_2H_4]$^+$),151([M+H-C_4H_8 –C_2H_2]$^+$)	sesquiterpene	AM

36	107.8	14-acetoxy-12-senecioyloxytetradeca-2E,8EZ,10E-trien-4,6-diyn-1-ol	$C_{21}H_{24}O_5$	379.4[M+Na]$^+$	—	279([M+Na-SenOH]$^+$),257([M+Na-SenONa]$^+$),197([M+Na-SenOH-AcONa]$^+$),179[M+Na-SenOH-AcONa-H_2O]$^+$	polyacetylene	AM
37	111.4	neocryptomerin	$C_{31}H_{20}O_{10}$	553.2	551.2	387(^{1,3}IB $^+$),286([Flavone II+OH]$^{+\bullet}$),270([Flavone II] $^{+\bullet}$), 268([Flavone I-O] $^{+\bullet}$),257 ([Flavone II+O-CO]$^+$)	biflavonoid	ST
38	112.2	14-acetoxy-12-senecioyloxytetradeca-2E,8EZ,10E-trien-4,6-diyn-1-ol	$C_{21}H_{24}O_5$	379.2 [M+Na]$^+$	393	279([M+Na-SenOH]$^+$),257([M+Na-SenONa]$^+$),197([M+Na-SenOH-AcONa]$^+$),179[M+Na-SenOH-AcONa-H_2O]$^+$	polyacetylene	AM
39	113.0	14-acetoxy-12-methylbutyryltetradeca-2E,8EZ,10E-trien-4, 6-diyn-1-ol	$C_{21}H_{26}O_5$	381.4 [M+Na]$^+$	—	279([M+Na-MeBOH]$^+$),257([M+Na-MeBONa]$^+$),197([M+Na-MeBOH-AcONa]$^+$),179[M+Na-MeBOH-AcONa-H_2O]$^+$	polyacetylene	AM
40	114.0	unknown						
41	115.6	14-acetoxy-12-methylbutyryltetradeca-2E,8EZ,10E-trien-4, 6-diyn-1-ol	$C_{21}H_{26}O_5$	381.4 [M+Na]	—	279([M+Na-MeBOH]$^+$),257([M+Na-MeBONa]$^+$),197([M+Na-MeBOH-AcONa]$^+$),179[M+Na-MeBOH-AcONa-H_2O]$^+$	polyacetylene	AM
42	116.2	unknown						
43	119.2	8-methoxyatractylenolide I	$C_{16}H_{22}O_3$	263.2	261.2	231([M+H-CH_3OH]$^+$), 213([M+H-CH_3OH-H_2O]$^+$), 203([M+H-CH_3OH-CO]$^+$),189([M+H-CH_3OH-C_3H_6]$^+$), 185([M+H-CH_3OH-H_2O-CO]$^+$), 163([M+H-CH_3OH-C_5H_8]$^+$)	sesquiterpene	AM

44	123.0	atractylenolide II*	$C_{15}H_{18}O_2$	231.4	229.4	213($[M+H-H_2O]^+$),203($[M+H-C_2H_4]^+$),185($[M+H-H_2O-CO]^+$), 157($[M+H-H_2O-CO-C_2H_4]^+$),143($[M+H-H_2O-CO-C_3H_6]^+$)	sesquiterpene	AM
45	124.2	unknown		496.4	—	478,313,258,284,104		AM
46	128.1	7,7”-di-*O*-methylhinokiflavone	$C_{32}H_{22}O_{10}$	567.6	565.4	401($^{1,3}IB^+$),299($[Flavone\ II+O]^+$),284 ($[Flavone\ I]^{+\bullet}$ or $[Flavone\ II]^{+\bullet}$), 268($[Flavone\ I-O]^{+\bullet}$),256($[Flavone\ I\ or\ II\ -CO]^{+\bullet}$	biflavonoid	ST
47	131.2	unknown		219.4		201,159,145,123,95		AM
48	132.8	atractylenolide VI	$C_{15}H_{22}$	203.2	—	161($[M+H-C_3H_6]^+$), 133($[M+H-C_3H_6-C_2H_4]^+$), 105($[M+H-C_3H_6-C_4H_8]^+$)	sesquiterpene	AM

* Compared with a reference standard.

Note: t_R, retention time; AM, *Atractylodes macrocephala*; ST, *Selaginella tamariscina*; CR*, Cinnamomi Ramulus*; RA, *Radix Aconiti lateralis Preparata.*

Figure S1. The fragmentation pathways of the identified compounds

- Peak 1 (neoline) 1
- Peak 2 (fuziline) 1
- Peak 3 (talatizamine) 1
- Peak 4 (14-benzoyl-10-OH-mesaconine) 1
- Peak 5 (benzoylmesaconine) 2
- Peak 6 (14-benzoylaconine) 2
- Peak 7 (14-benzoylhypaconine) 2
- Peak 9 (10-OH-mesaconitine) 3
- Peak 10 (14-benzoyldeoxyaconine) 3
- Peak 11 (mesaconitine) 3
- Peak 12 (10-OH-aconitine) 4
- Peak 13 (coumarin) 4
- Peak 14 (hypaconitine) 4
- Peak 15 (aconitine) 5
- Peak 16 (13-deoxyhypaconitine) 5
- Peak 17 (2-hydroxy cinnamaldehyde) 5
- Peak 18 (deoxyaconitine) 6
- Peak 19 (cinnamic alcohol) 6
- Peak 20 (cinnamic acid) 6
- Peak 21 (cinnamic aldehyde) 6
- Peak 22 (selaginellin) 6
- Peak 23 (2-methoxy cinnamaldehyde) 7
- Peak 24 (amentoflavone) 7
- Peak 25 (2″,3″-dihydroamentoflavone) 8
- Peak 26 (2,3-dihydrorobustaflavone) 8
- Peak 27 (robustaflavone) 9
- Peak 28 (7″-O-methylamentoflavone) 9
- Peak 29 (atractylenolide III) 11
- Peak 30 (7-O-methylamentoflavone) 13
- Peak 31 (hinokiflavone) 14
- Peak 33 (isocryptomerin) 15
- Peaks 34 and 35 (isoatracytlenolide I and atractylenolide I) 15
- Peaks 36 and 38 (14-acetoxy-12-senecioyloxytetradeca-2E,8EZ,10E-trien-4,6-diyn-1-ol) ...17
- Peak 37 (neocryptomerin) 18
- Peaks 39 and 41 (14-acetoxy-12-methylbutyryltetradeca-2E,8EZ,10E-trien-4,6-diyn-1-ol)..20
- Peaks 43 (8-methoxyatractylenolide I) 21
- Peak 44 (atractylenolide II) 22
- Peak 46 (7,7″-di-*O*-methylhinokiflavone) 23
- Peak 48 (atractylenolide VI) 24

- Peak 1 (neoline)

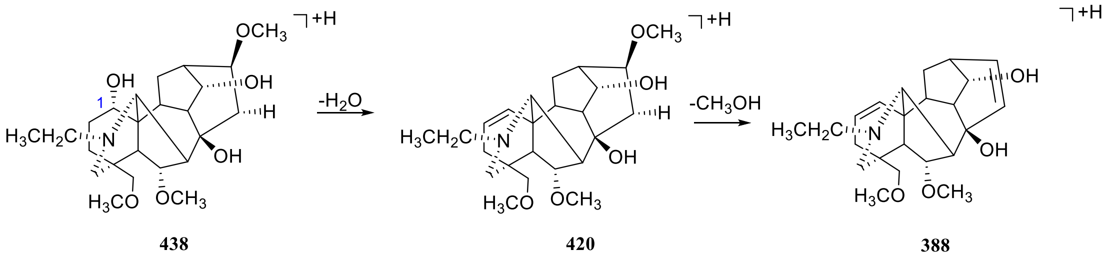

- Peak 2 (fuziline)

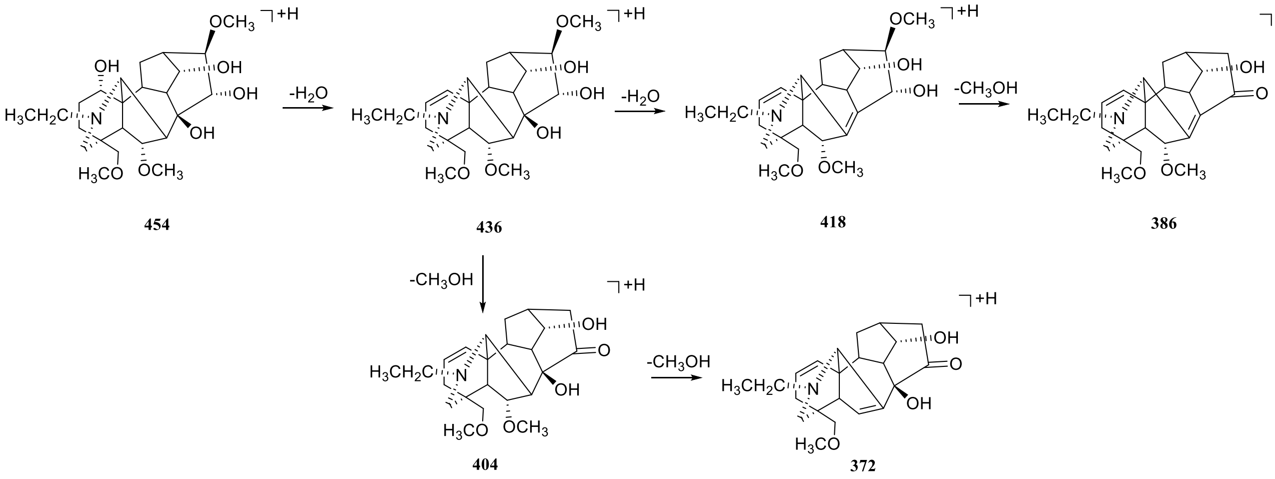

- Peak 3 (talatizamine)

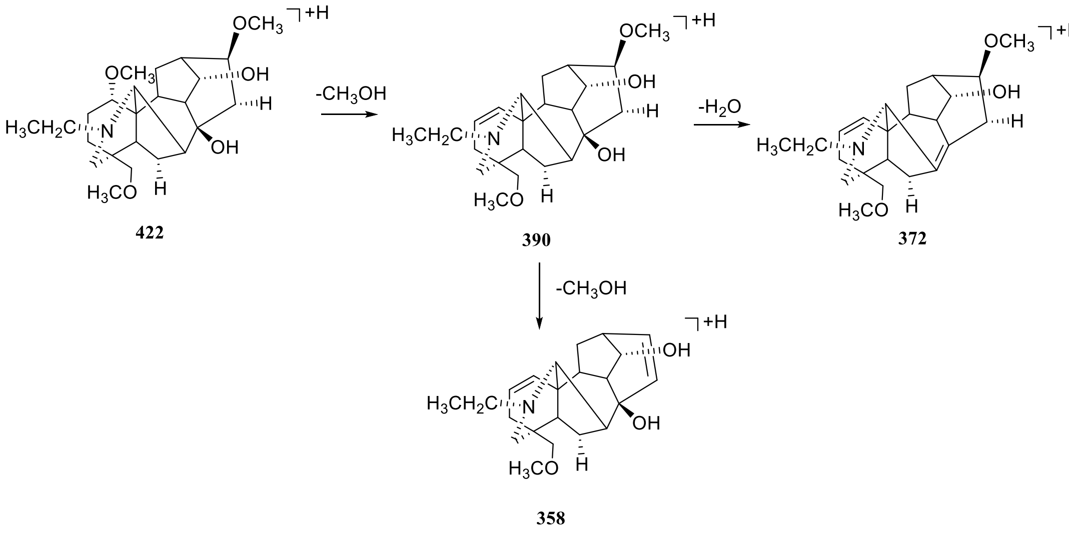

- Peak 4 (14-benzoyl-10-OH-mesaconine)

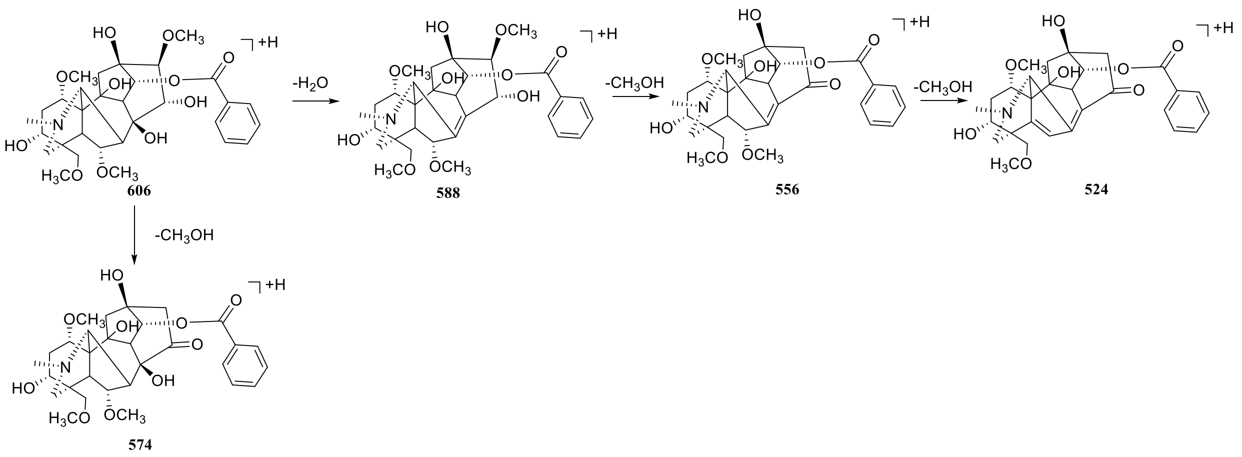

- Peak 5 (benzoylmesaconine)

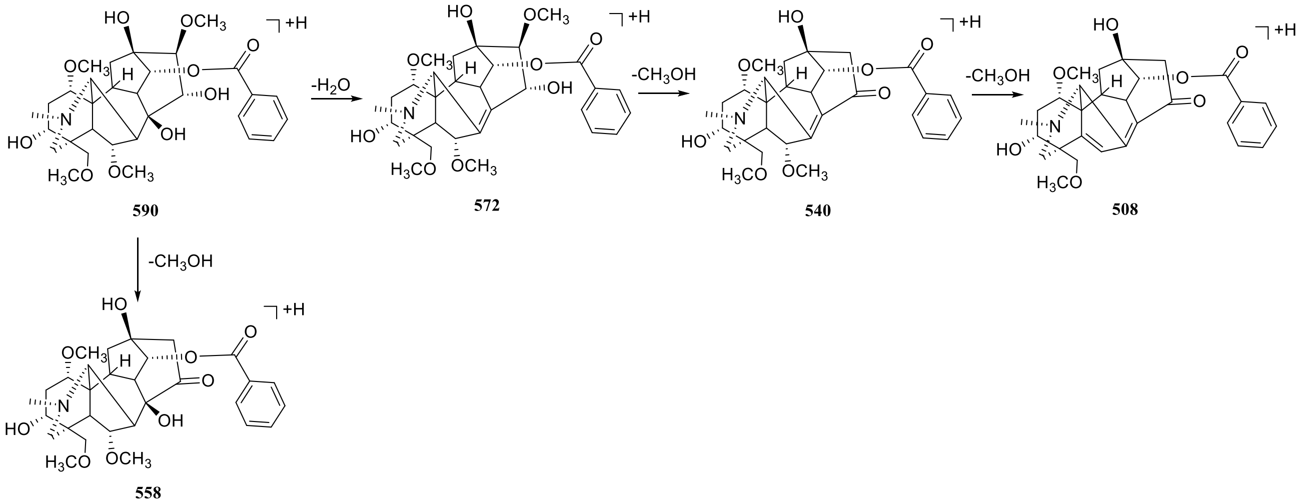

- Peak 6 (14-benzoylaconine)

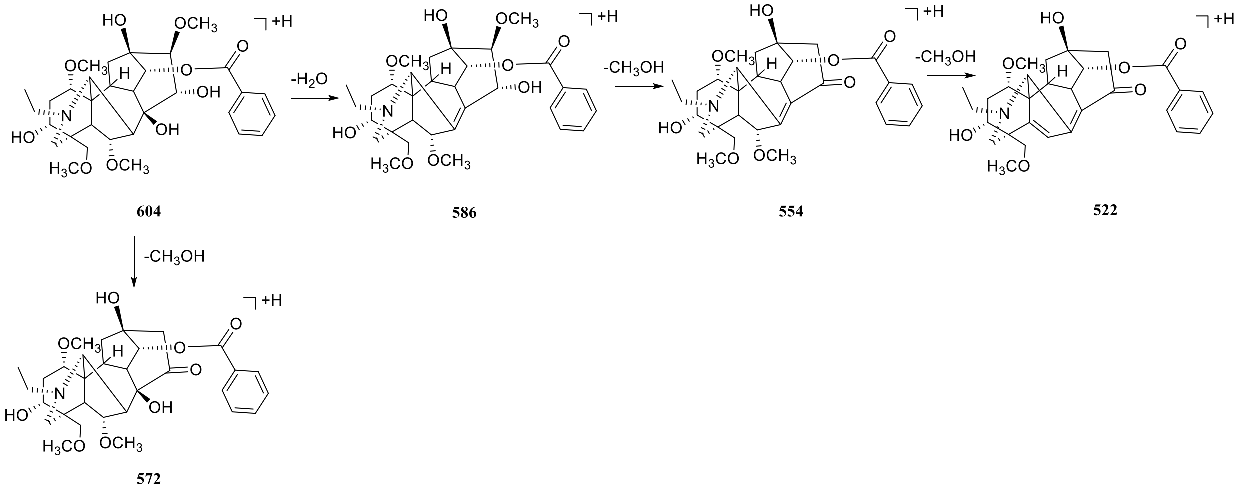

- Peak 7 (14-benzoylhypaconine)

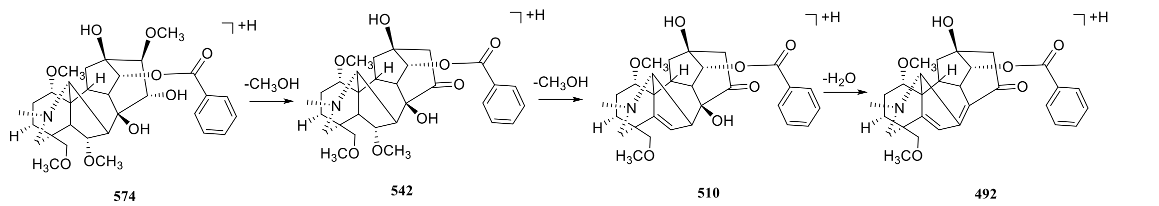

- Peak 9 (10-OH-mesaconitine)

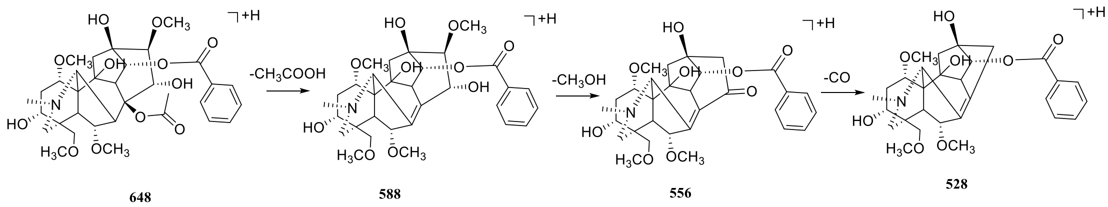

- Peak 10 (14-benzoyldeoxyaconine)

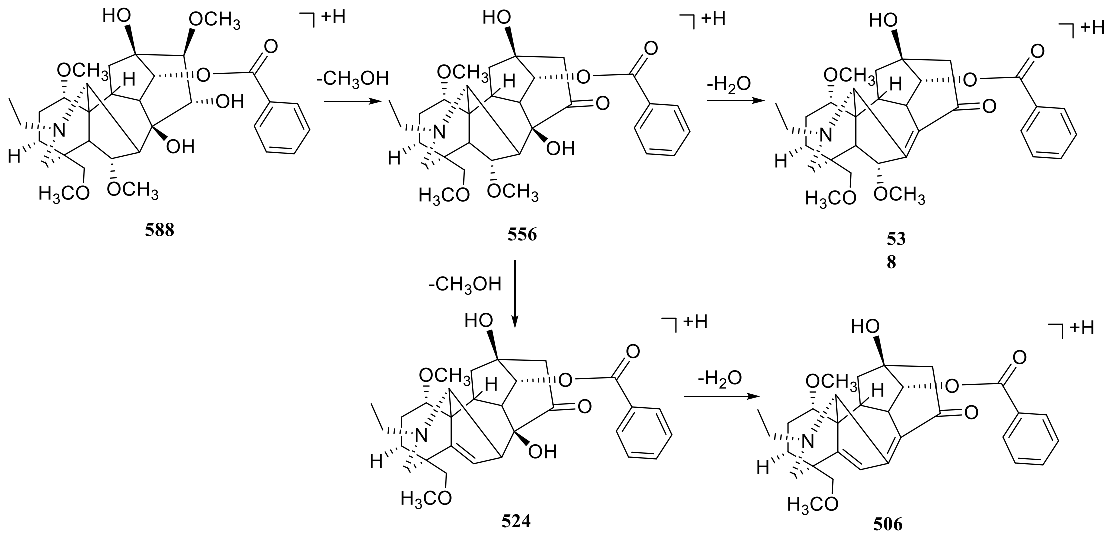

- Peak 11 (mesaconitine)

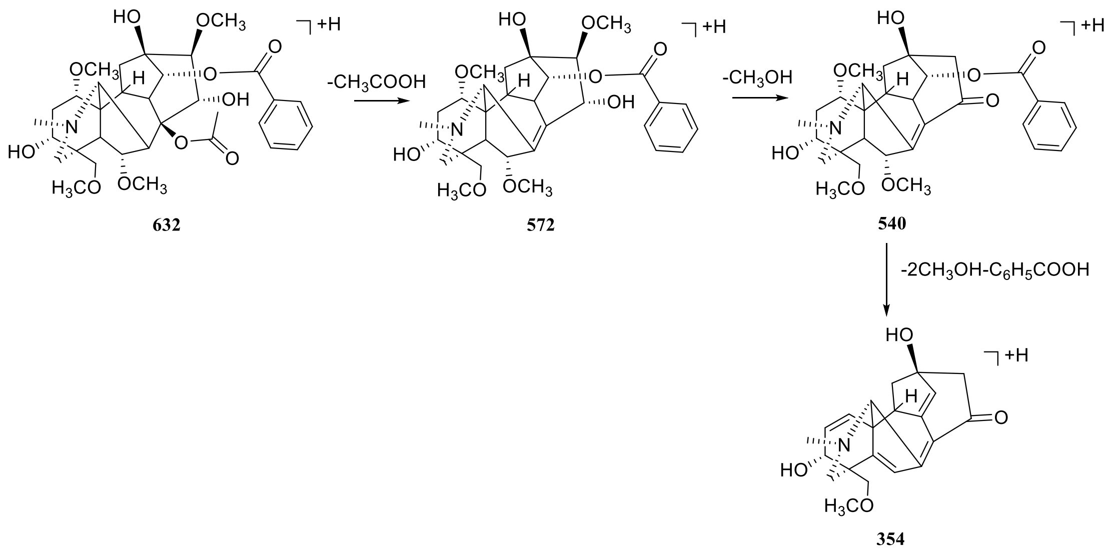

- Peak 12 (10-OH-aconitine)

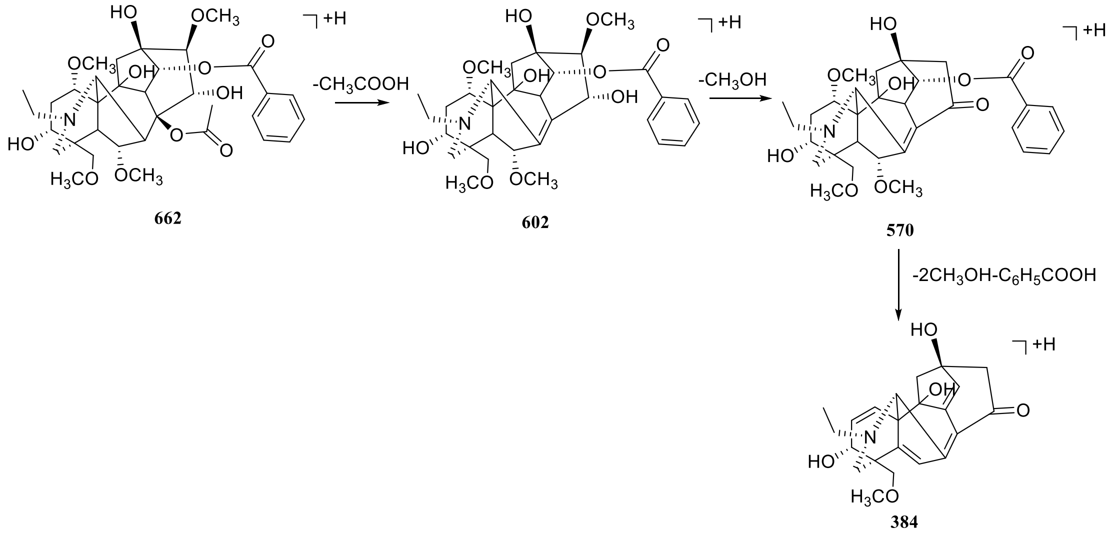

- Peak 13 (coumarin)

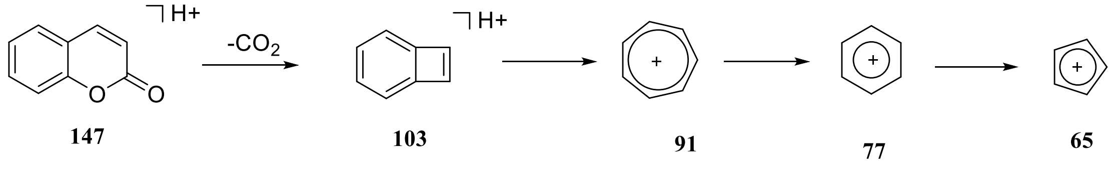

- Peak 14 (hypaconitine)

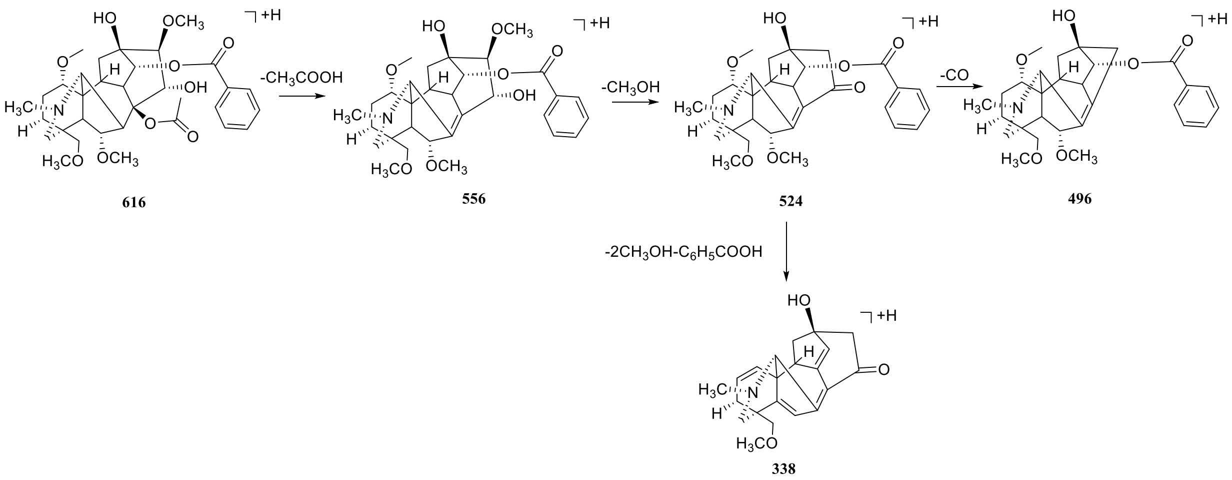

- Peak 15 (aconitine)

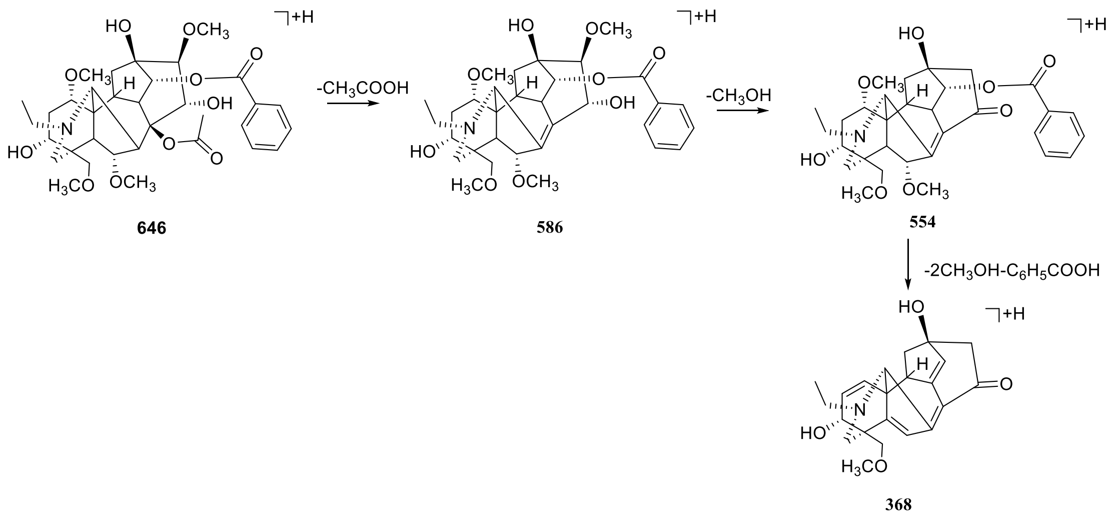

- Peak 16 (13-deoxyhypaconitine)

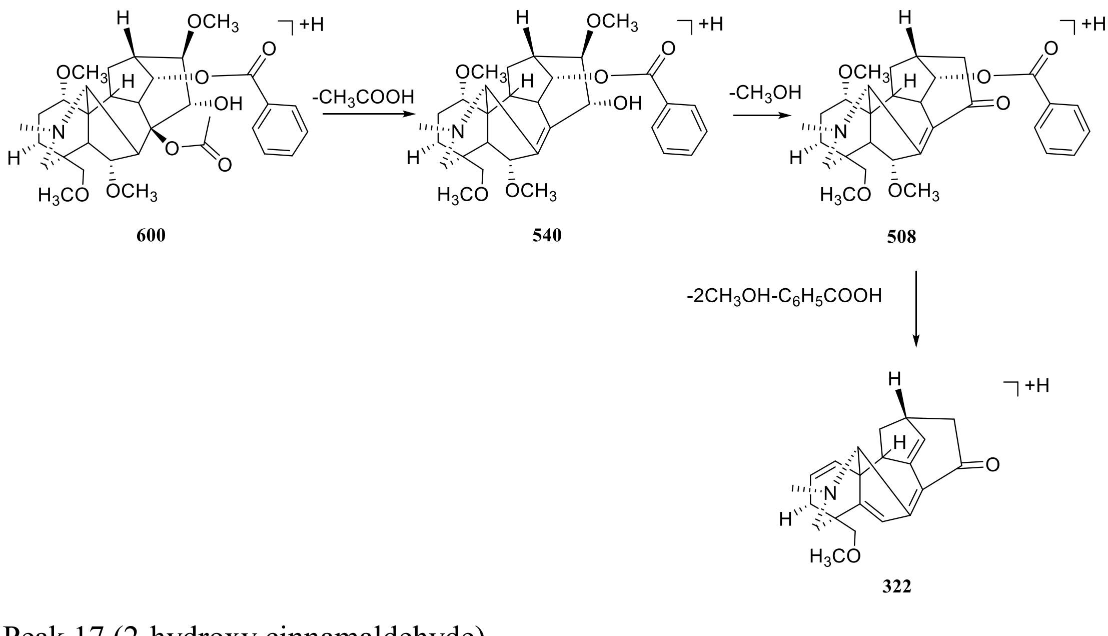

- Peak 17 (2-hydroxy cinnamaldehyde)

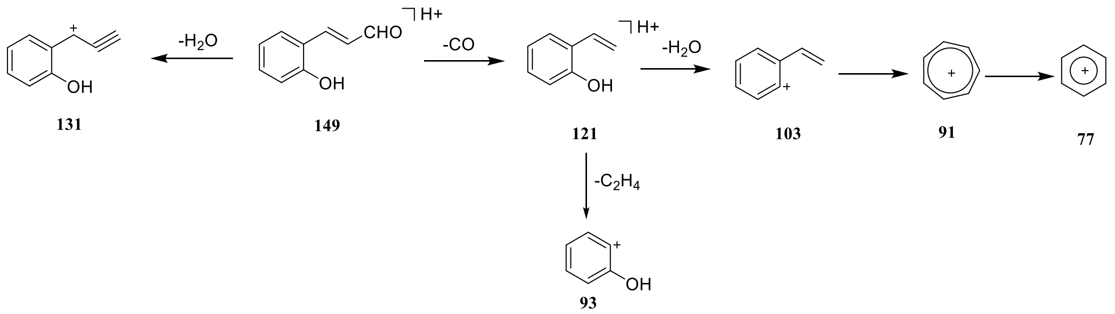

- Peak 18 (deoxyaconitine)

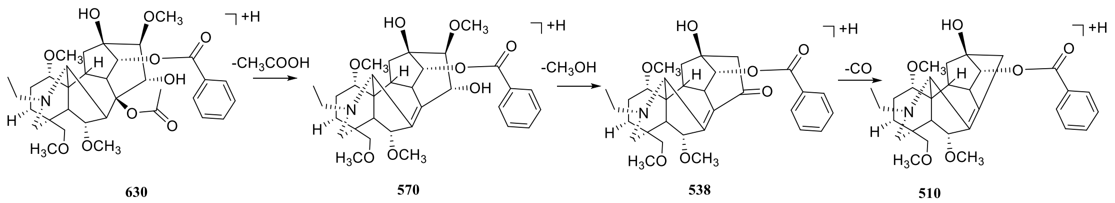

- Peak 19 (cinnamic alcohol)

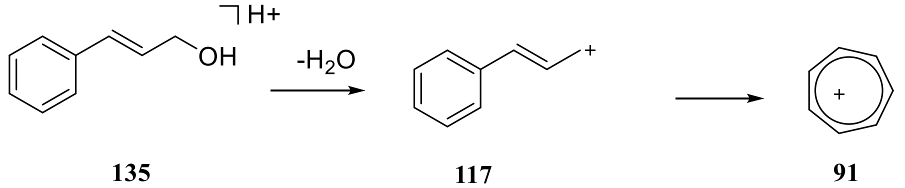

- Peak 20 (cinnamic acid)

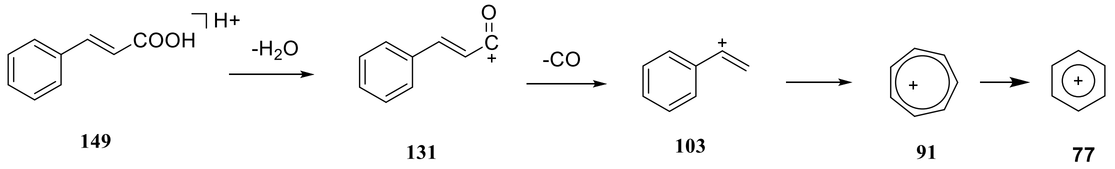

- Peak 21 (cinnamic aldehyde)

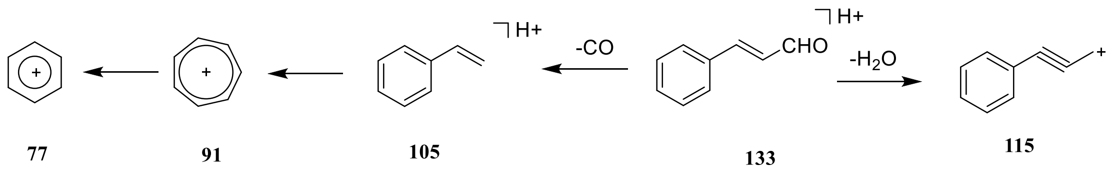

- Peak 22 (selaginellin)

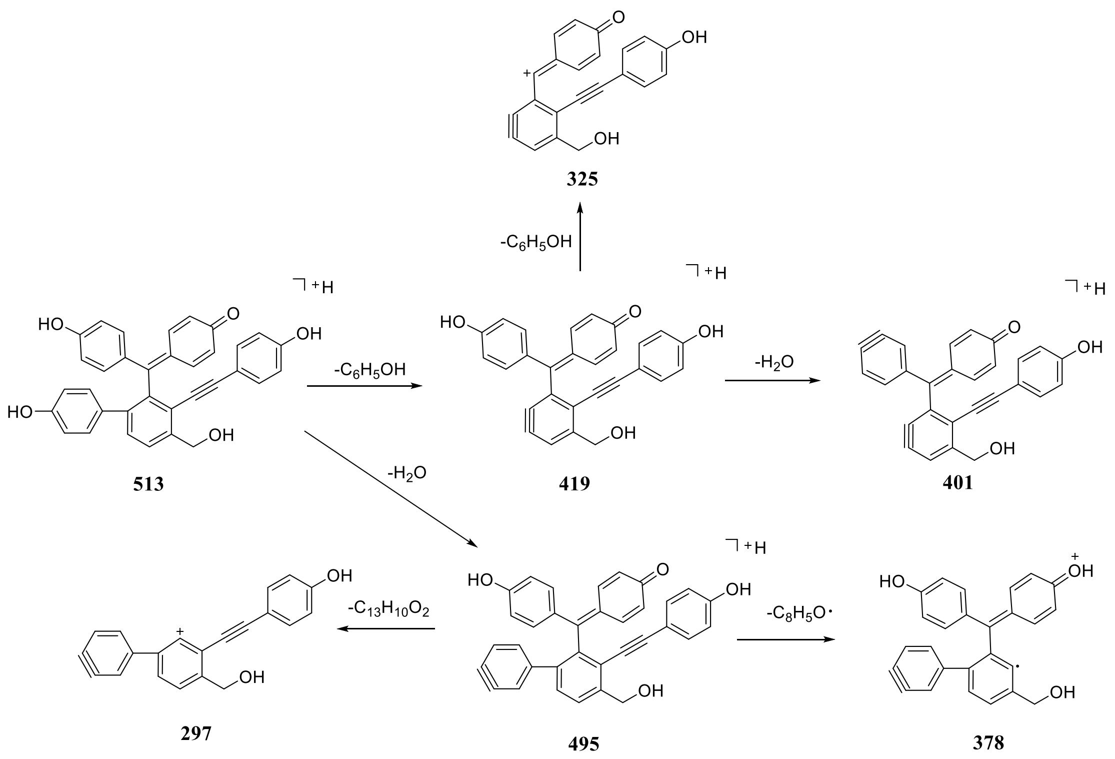

- Peak 23 (2-methoxy cinnamaldehyde)

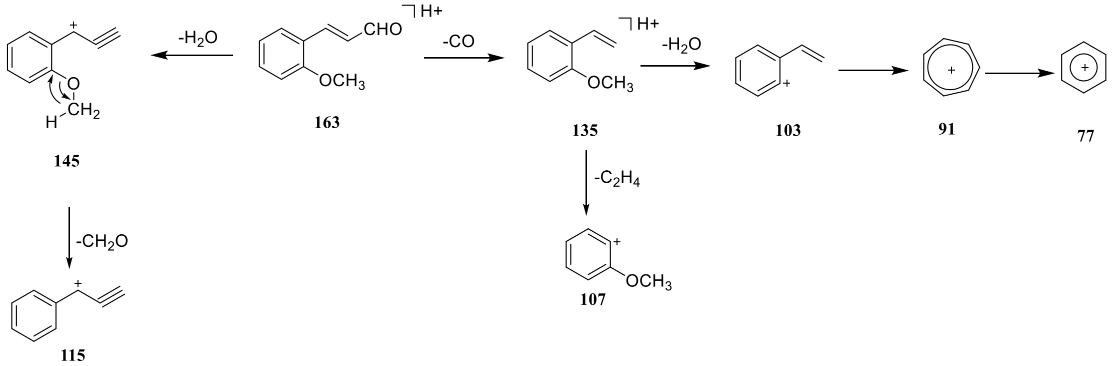

- Peak 24 (amentoflavone)

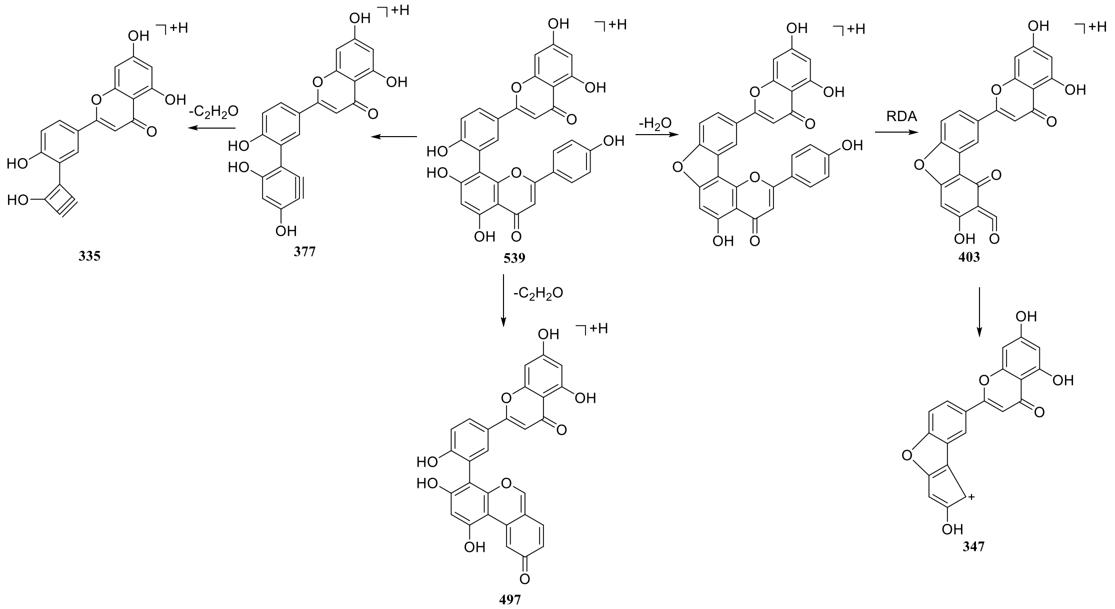

- Peak 25 (2″,3″-dihydroamentoflavone)

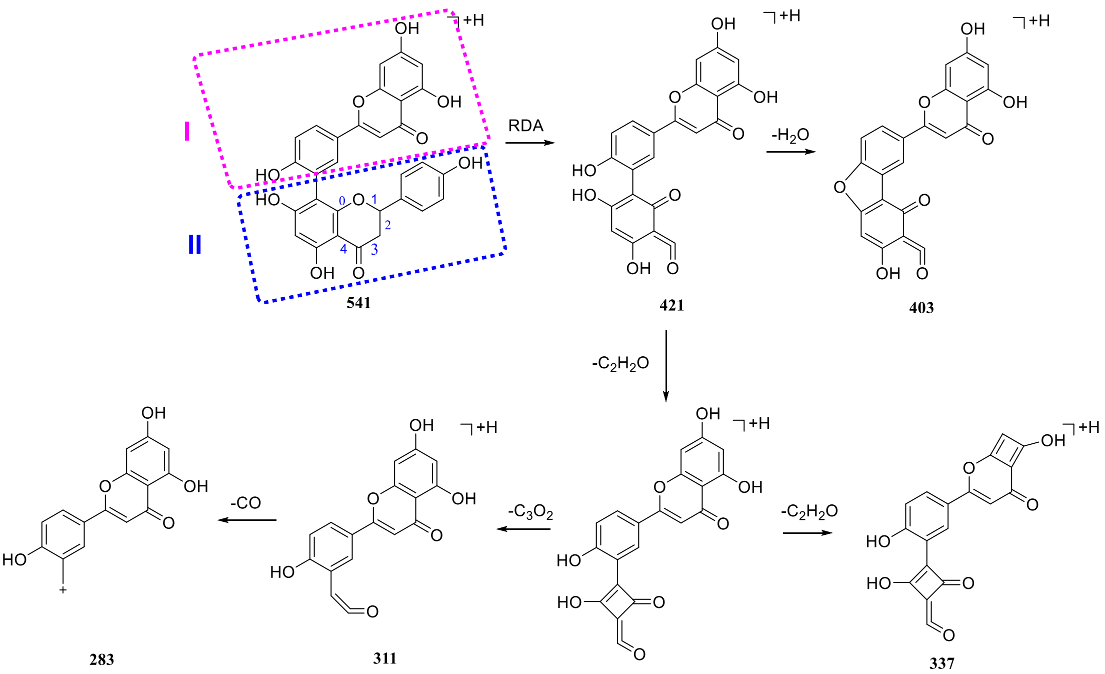

- Peak 26 (2,3-dihydrorobustaflavone)

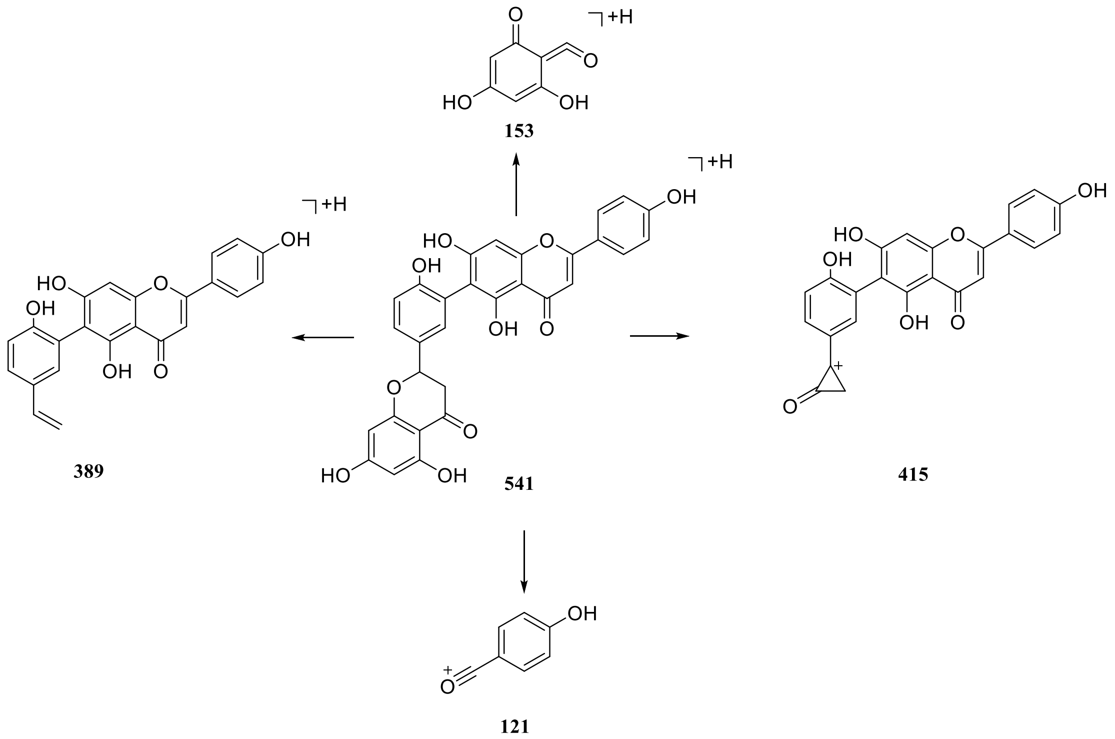

- Peak 27 (robustaflavone)

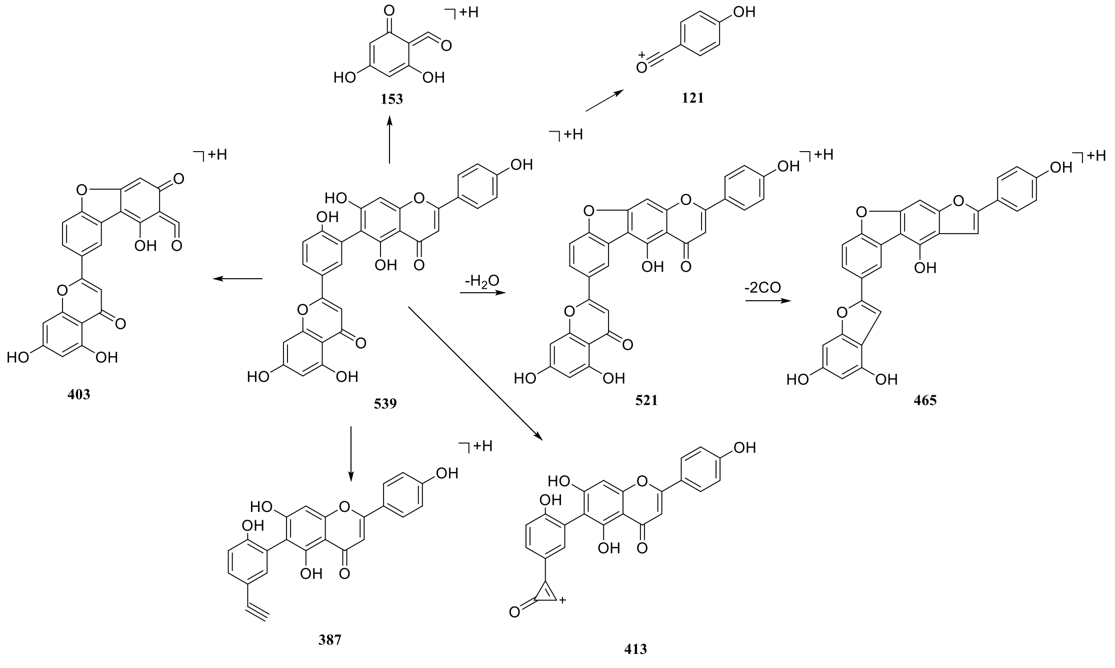

- Peak 28 (7″-O-methylamentoflavone)

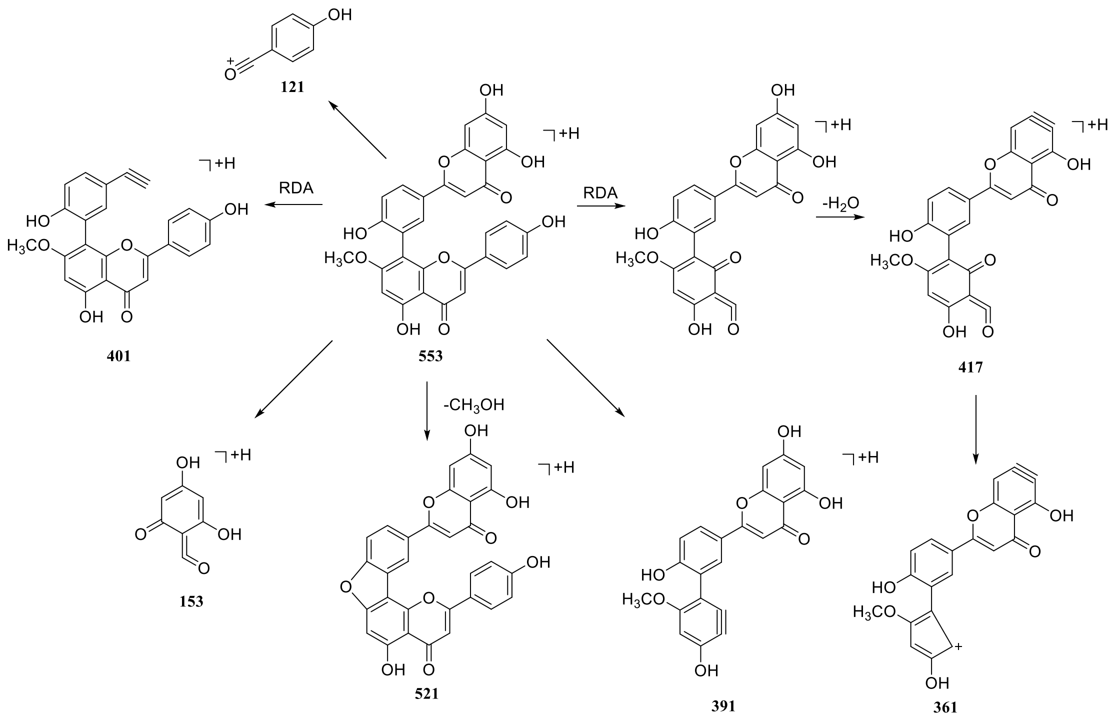
121
401
553
417
153
521
391
361
RDA
RDA
-H2O
-CH3OH
+H
OH
HO
H3CO
O

- Peak 29 (atractylenolide III)

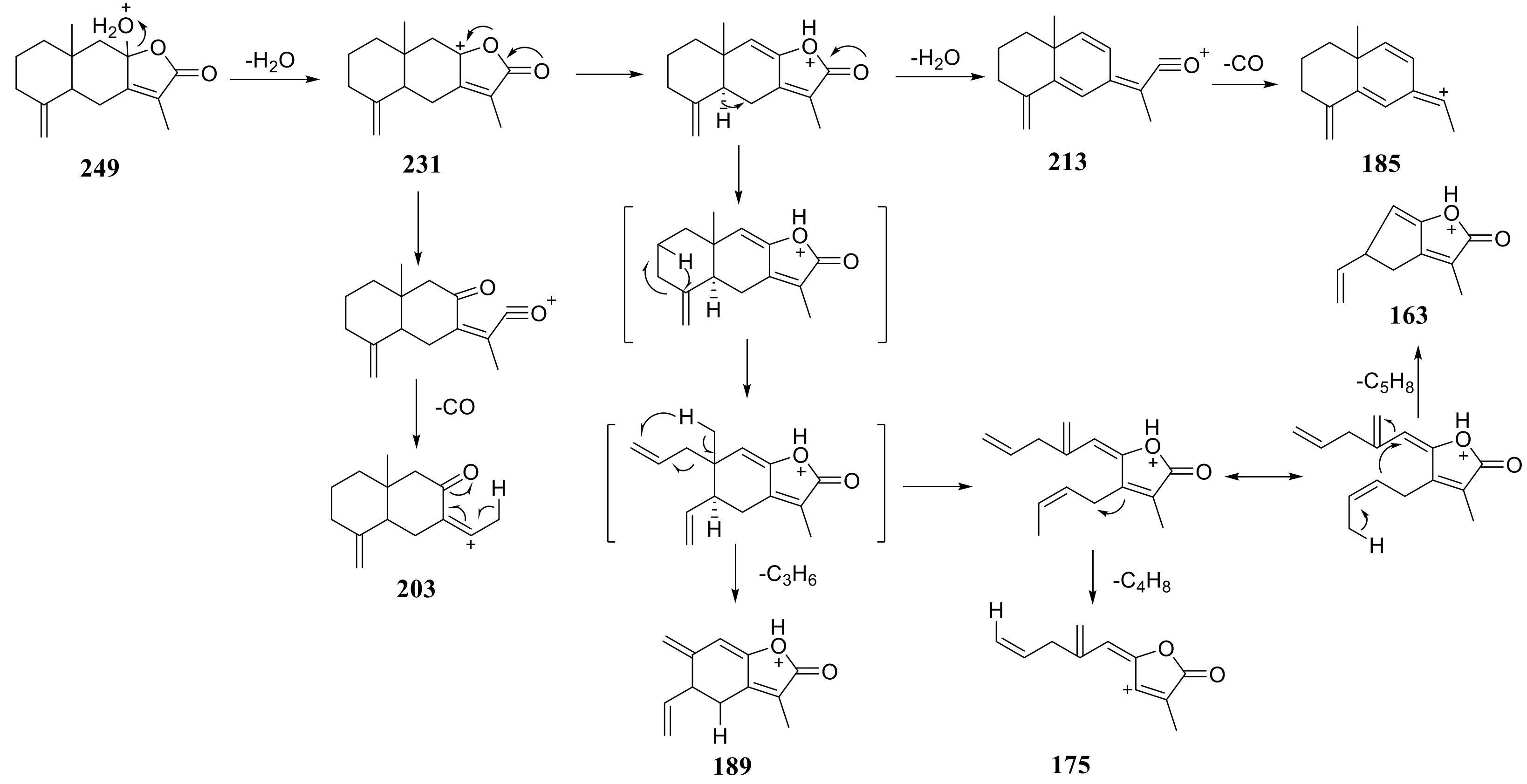

The fragmentation pathway of atractylenolide III were analyzed using a high-resolution linear ion trap Orbitrap mass spectrometer

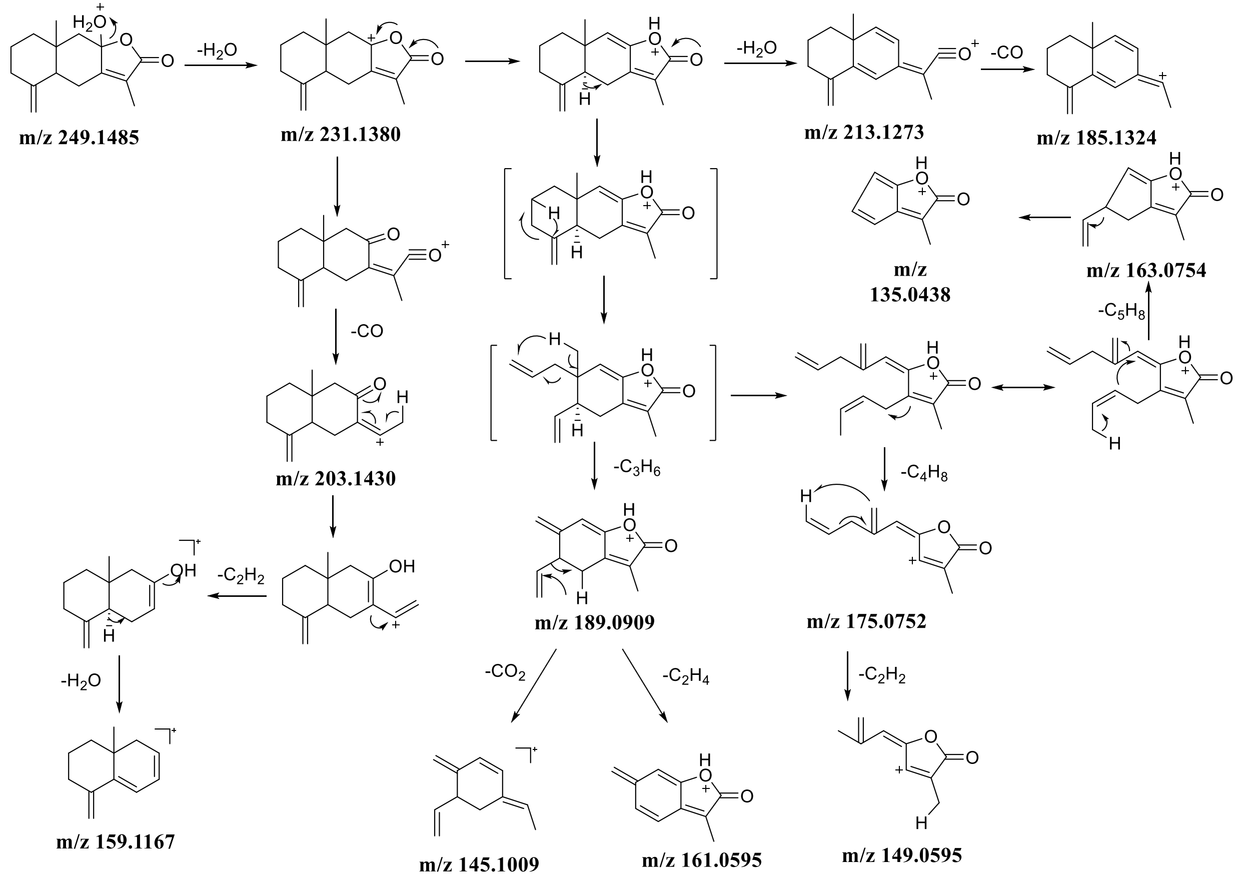

- Peak 30 (7-O-methylamentoflavone)

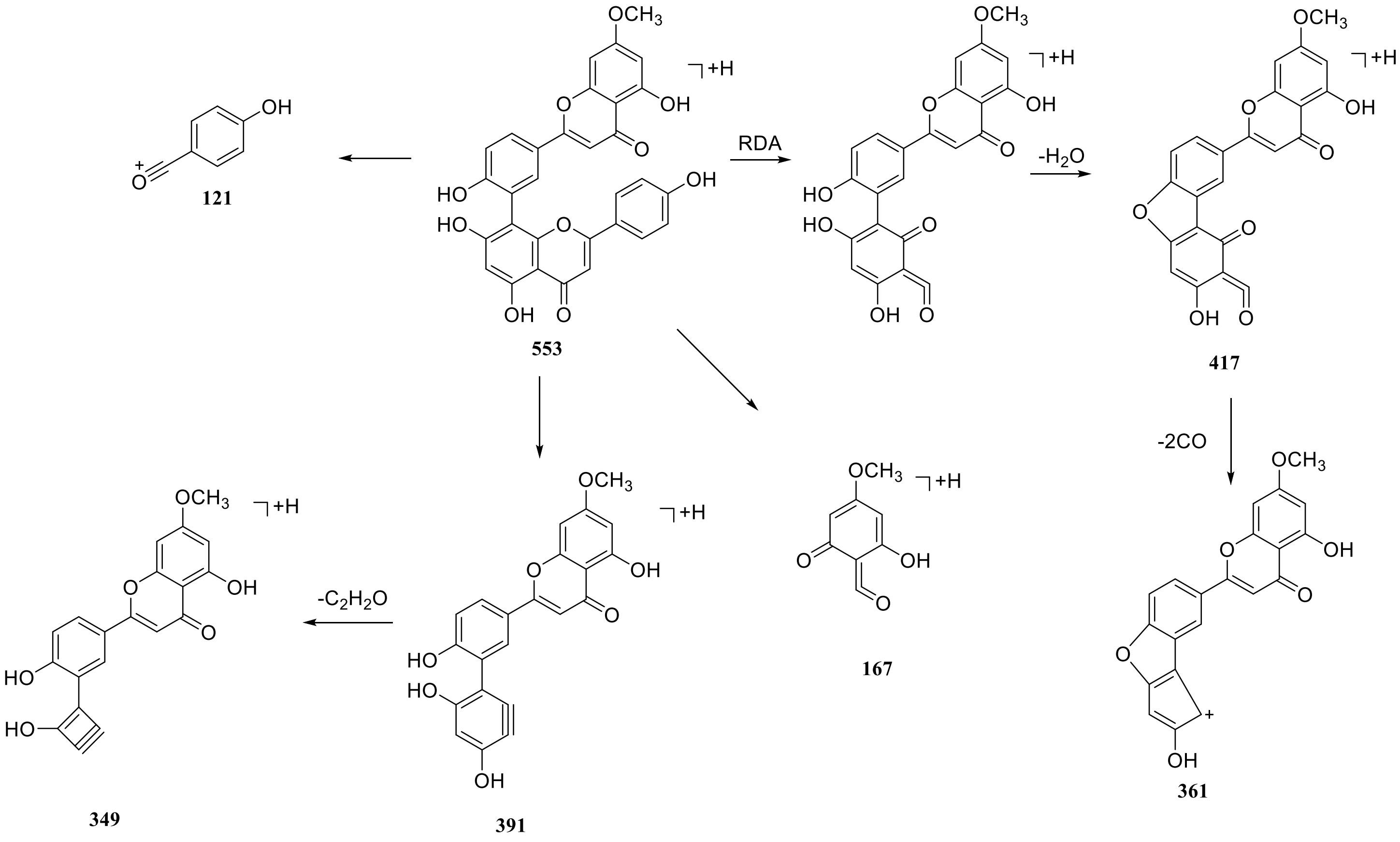

- Peak 31 (hinokiflavone)

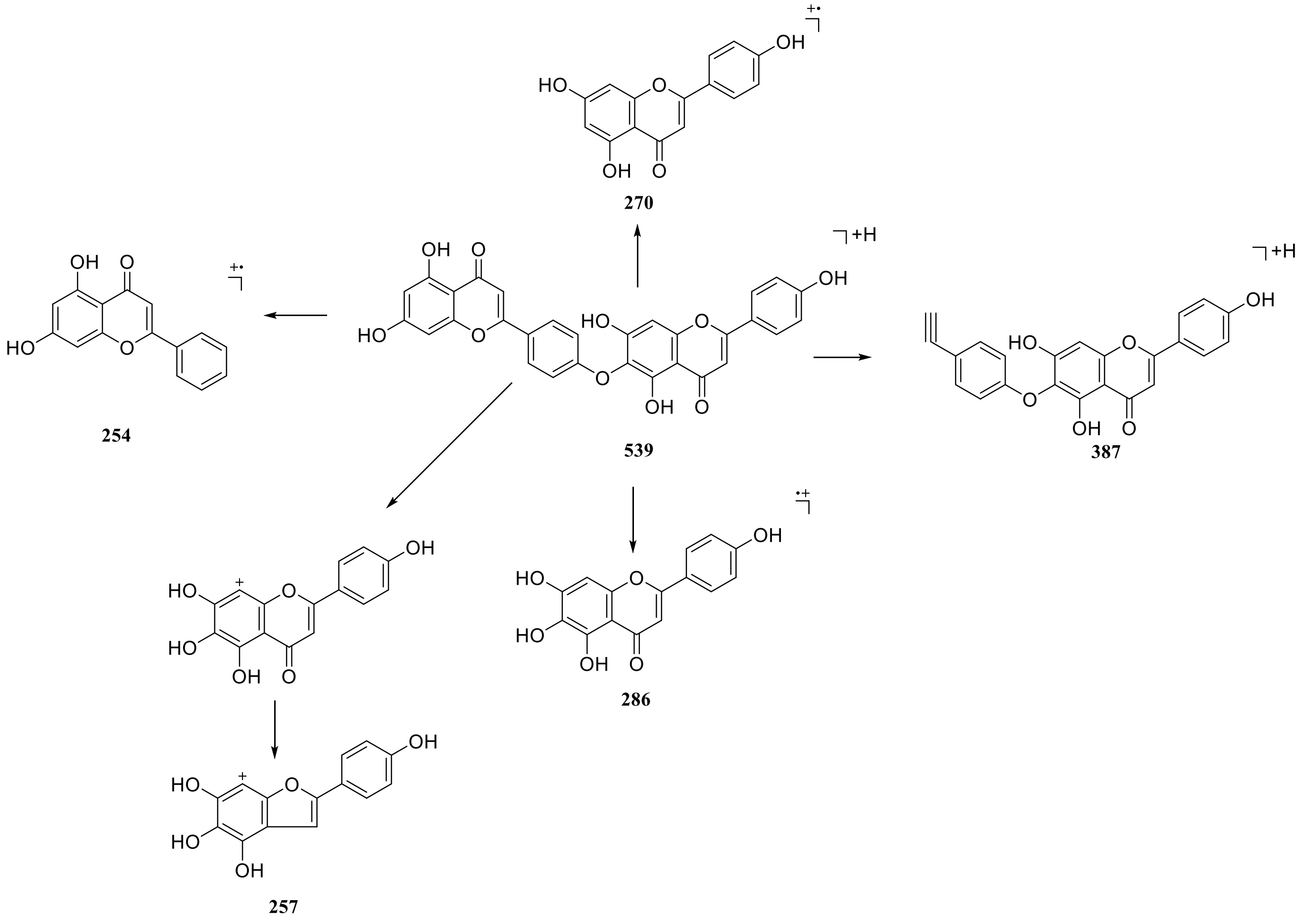

- Peak 33 (isocryptomerin)

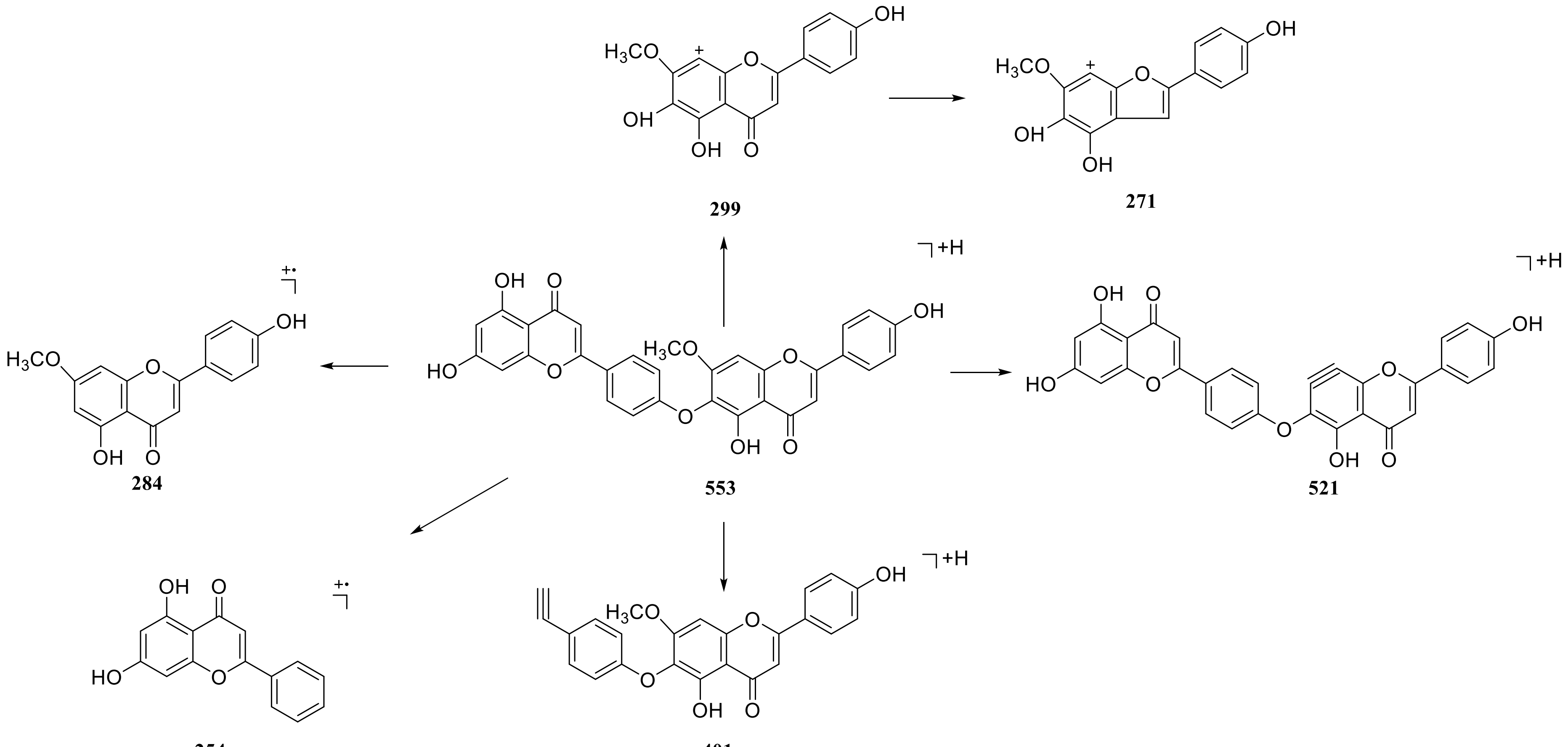

- Peaks 34 and 35 (isoatractylenolide I and atractylenolide I)

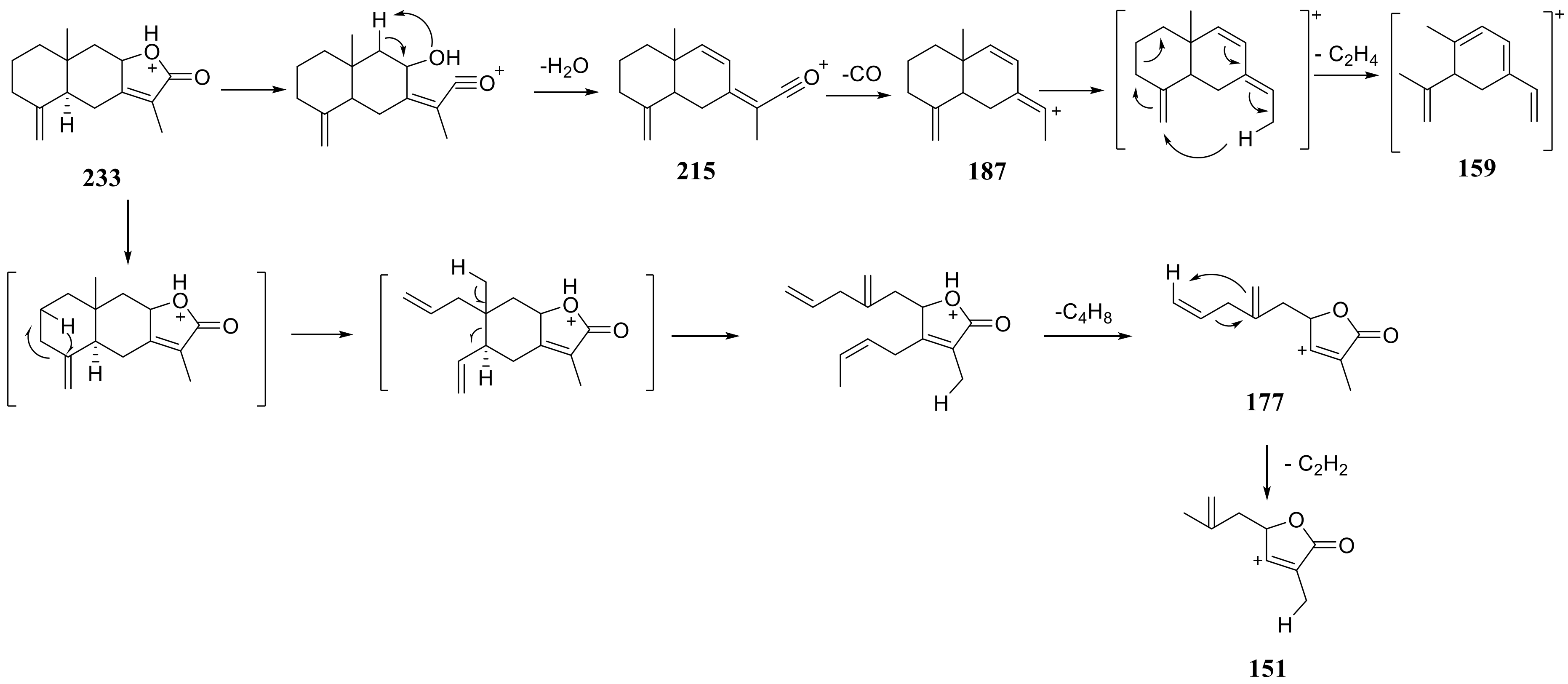

The fragmentation pathway of atractylenolide I were analyzed using a high-resolution linear ion trap Orbitrap mass spectrometer

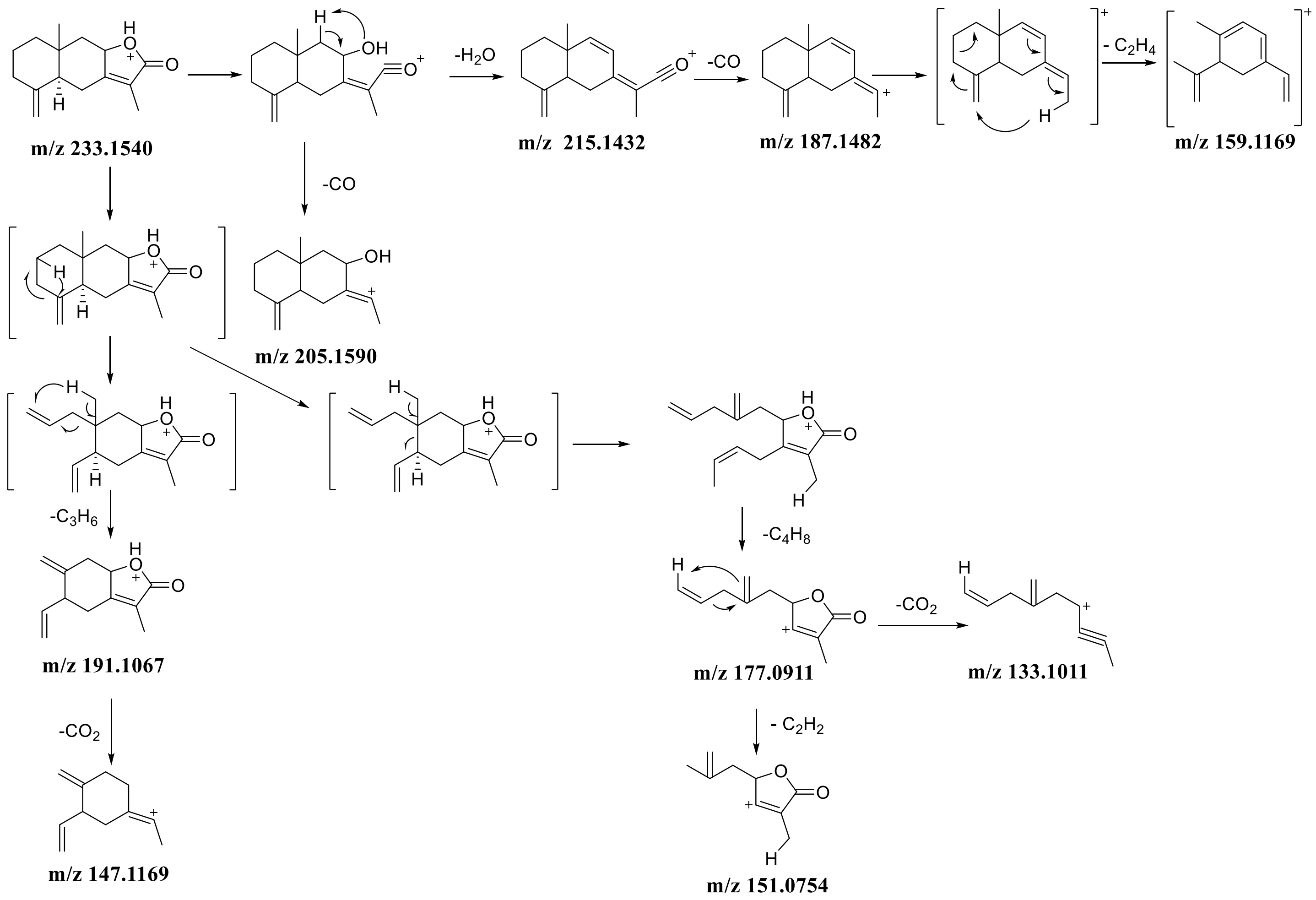

- Peaks 36 and 38 (14-acetoxy-12-senecioyloxytetradeca-2E,8EZ,10E-trien-4,6-diyn-1-ol)

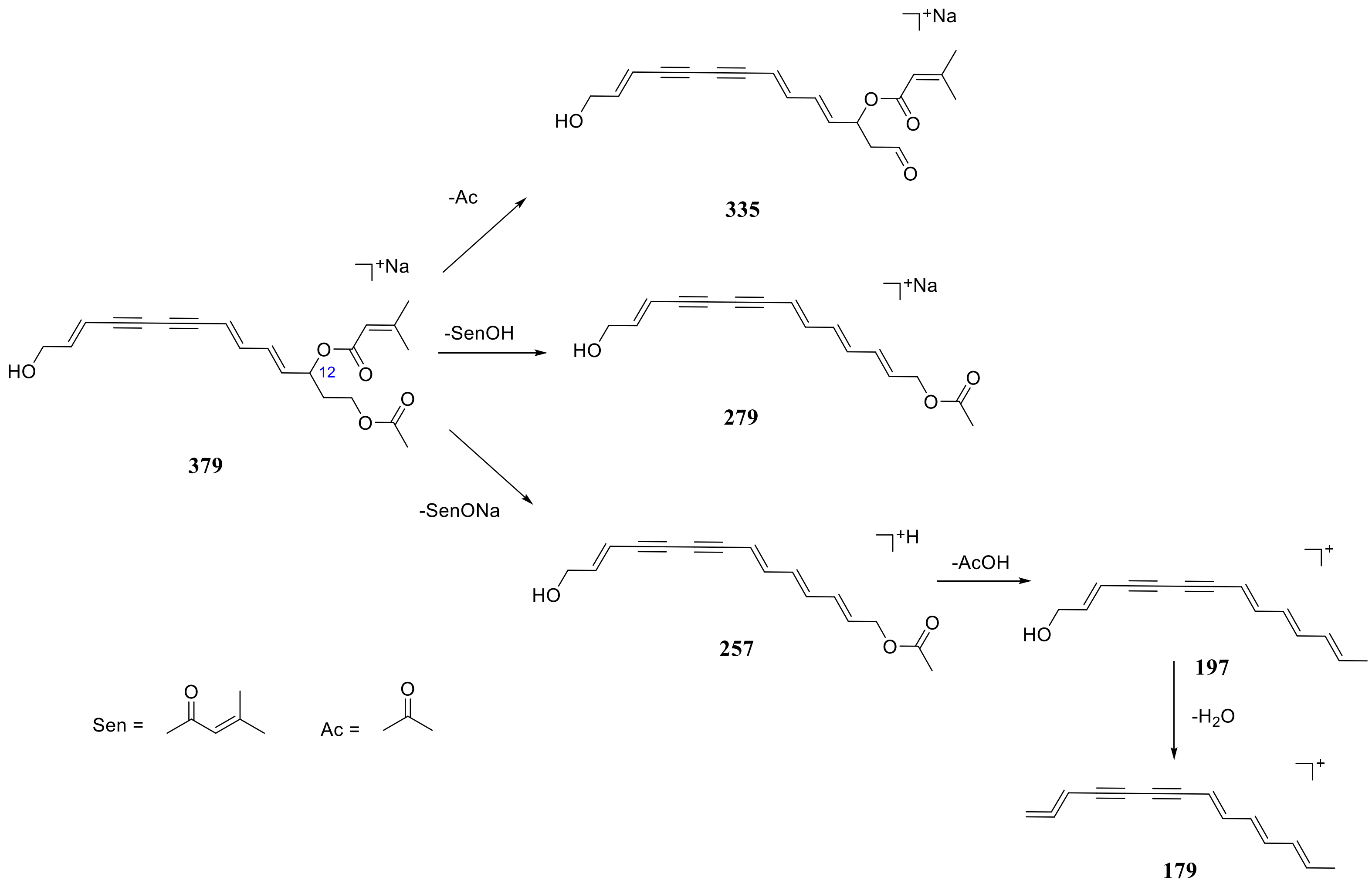

- Peak 37 (neocryptomerin)

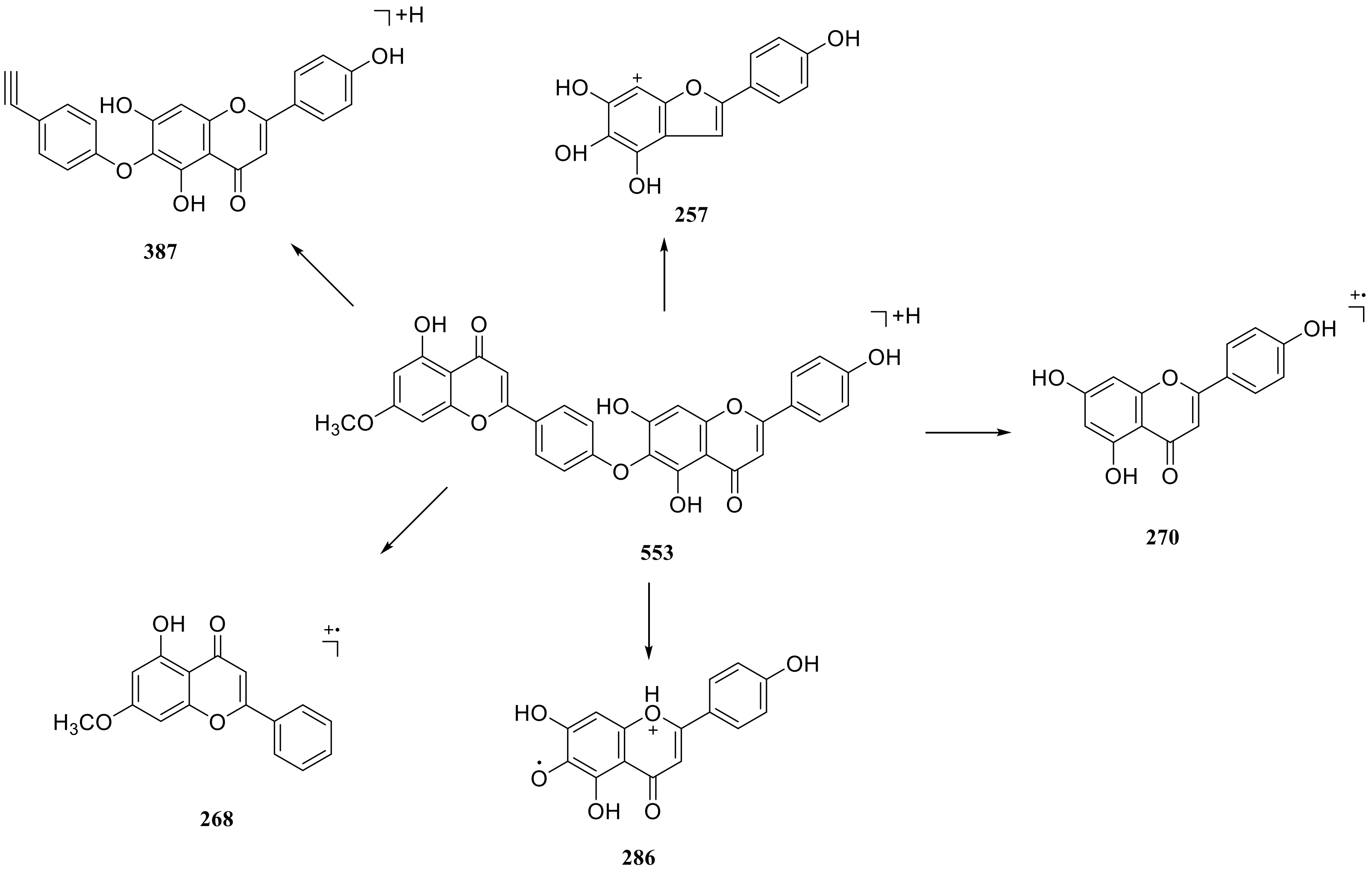
387
257
553
270
268
286

- Peaks 39 and 41 (14-acetoxy-12-methylbutyryltetradeca-2E,8EZ,10E-trien-4,6-diyn-1-ol)

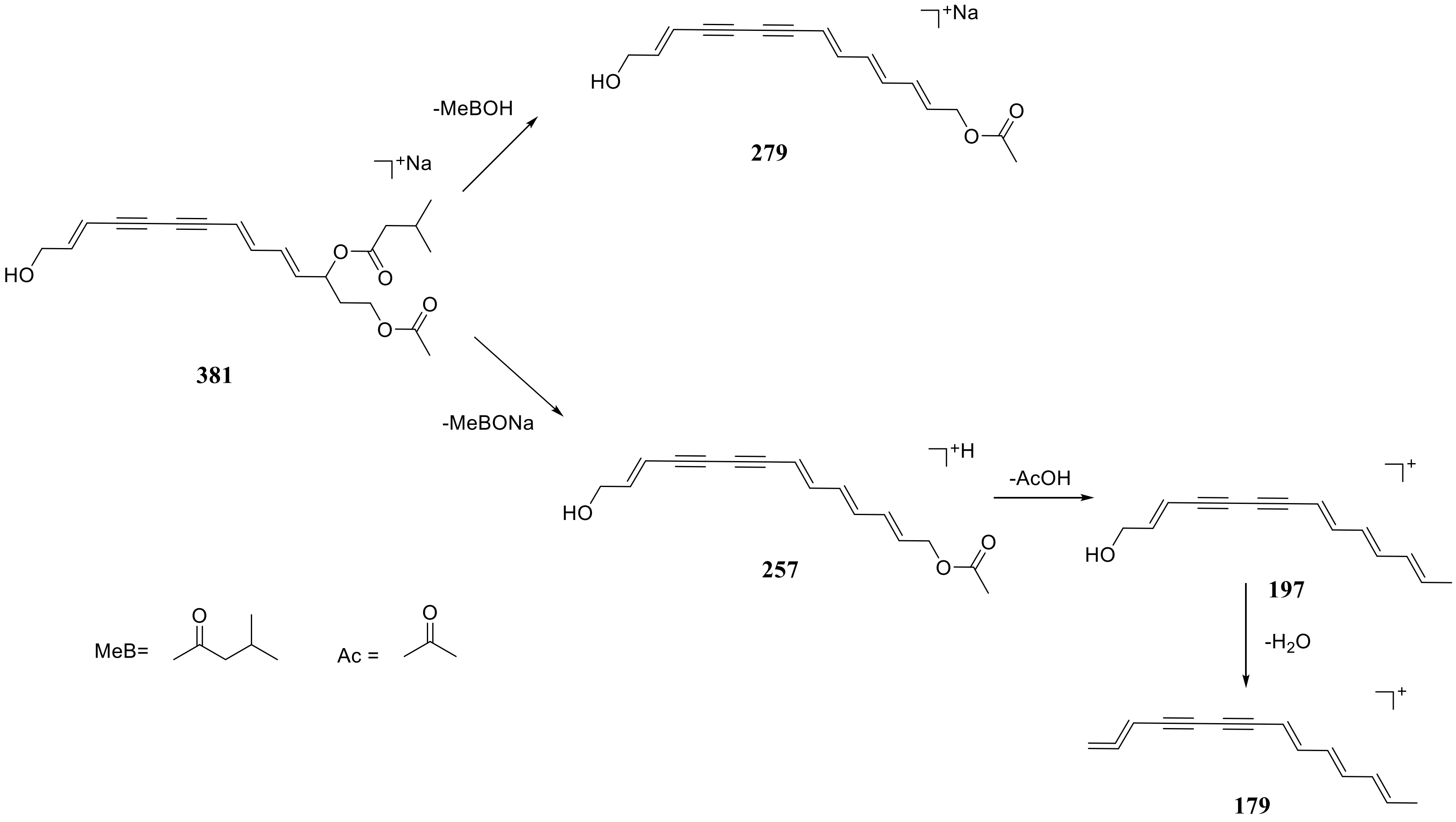

- Peaks 43 (8-methoxyatractylenolide I)

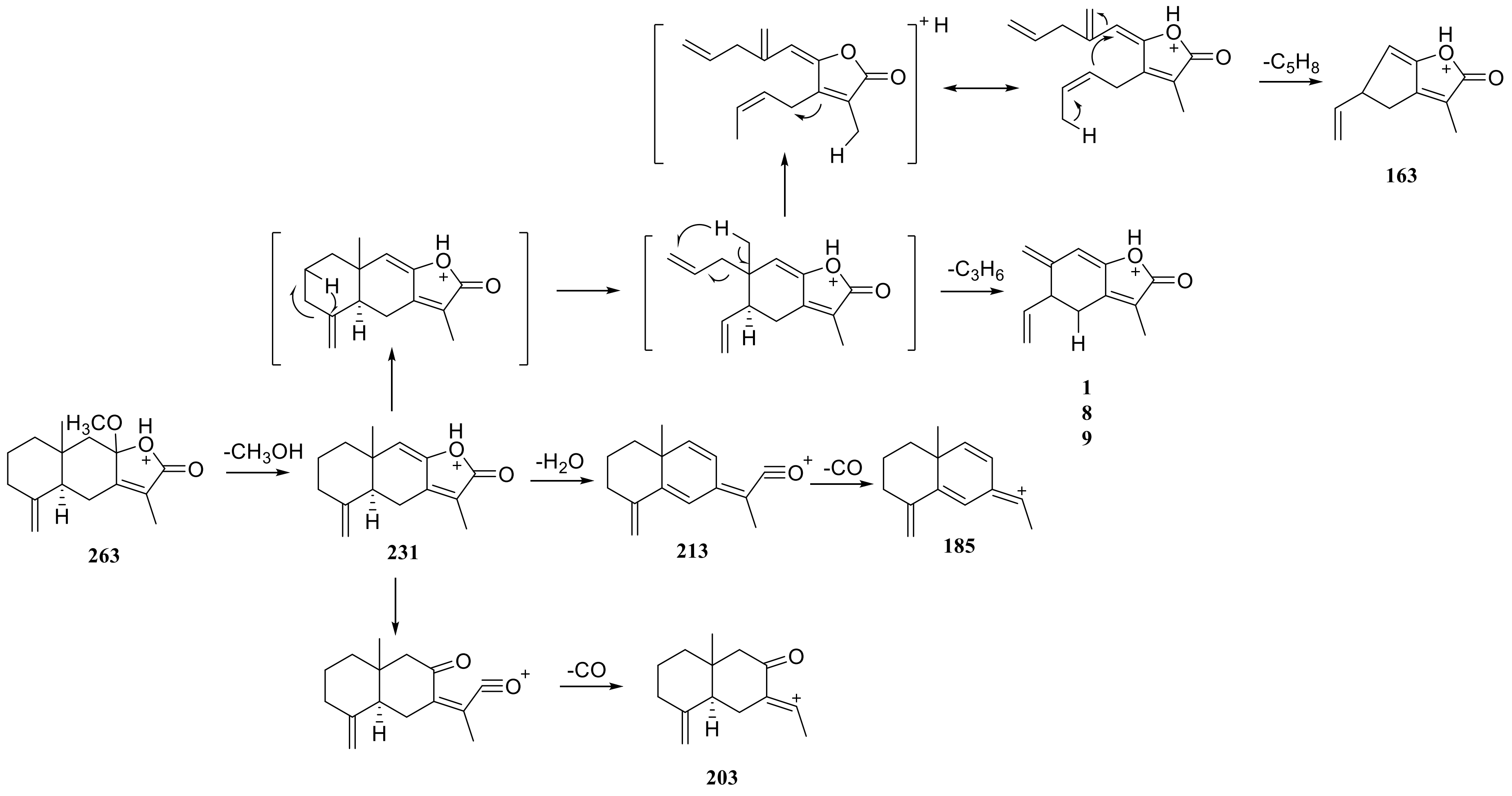

- Peak 44 (atractylenolide II)

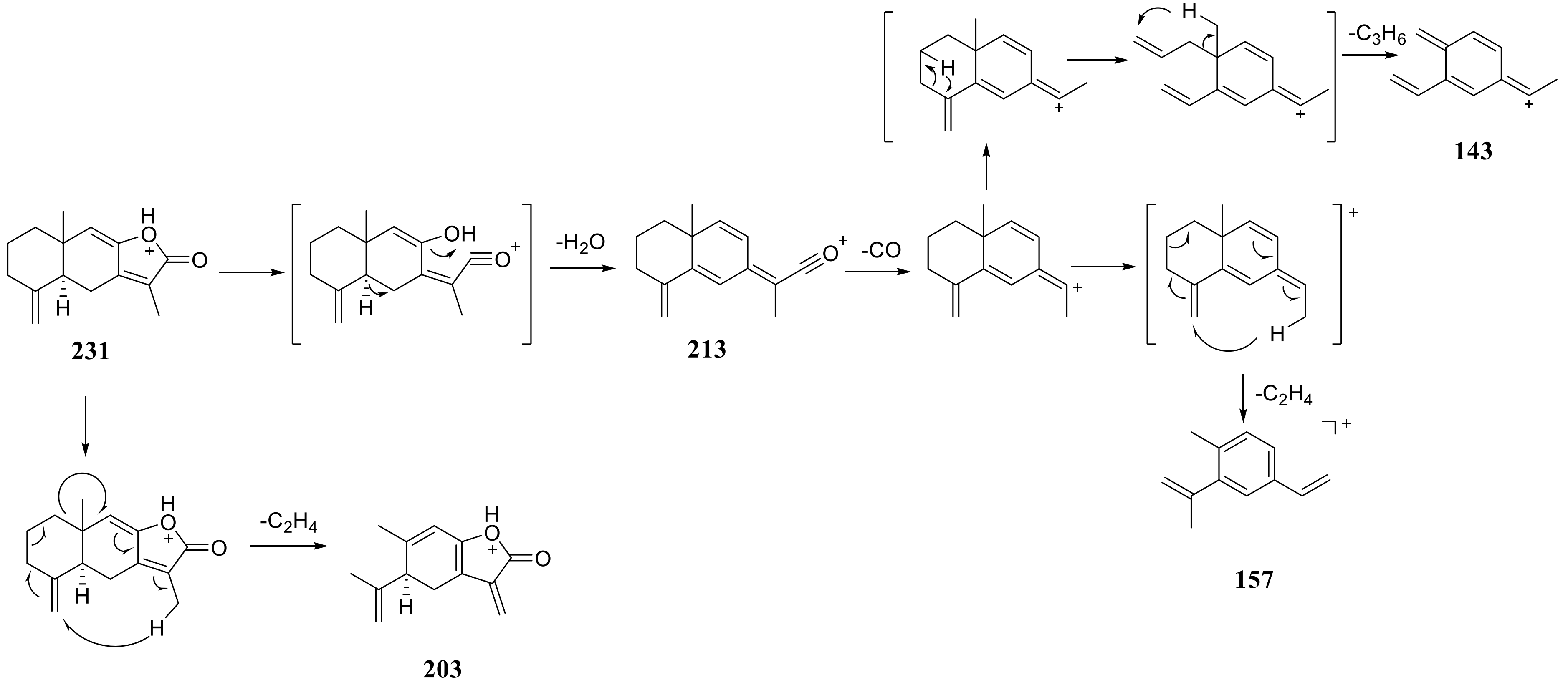

The fragmentation pathway of atractylenolide II were analyzed using a high-resolution linear ion trap Orbitrap mass spectrometer

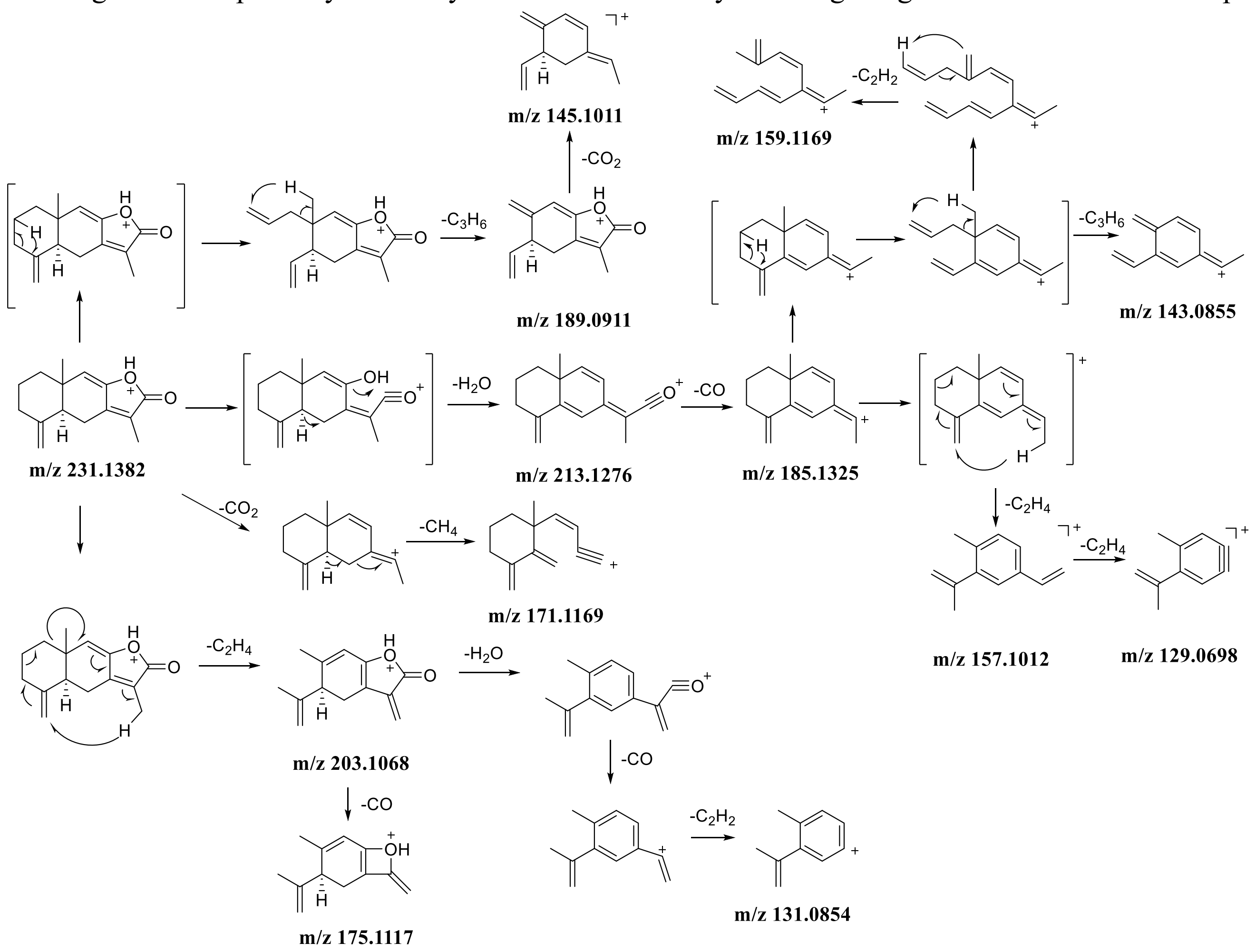

- Peak 46 (7,7″-di-*O*-methylhinokiflavone)

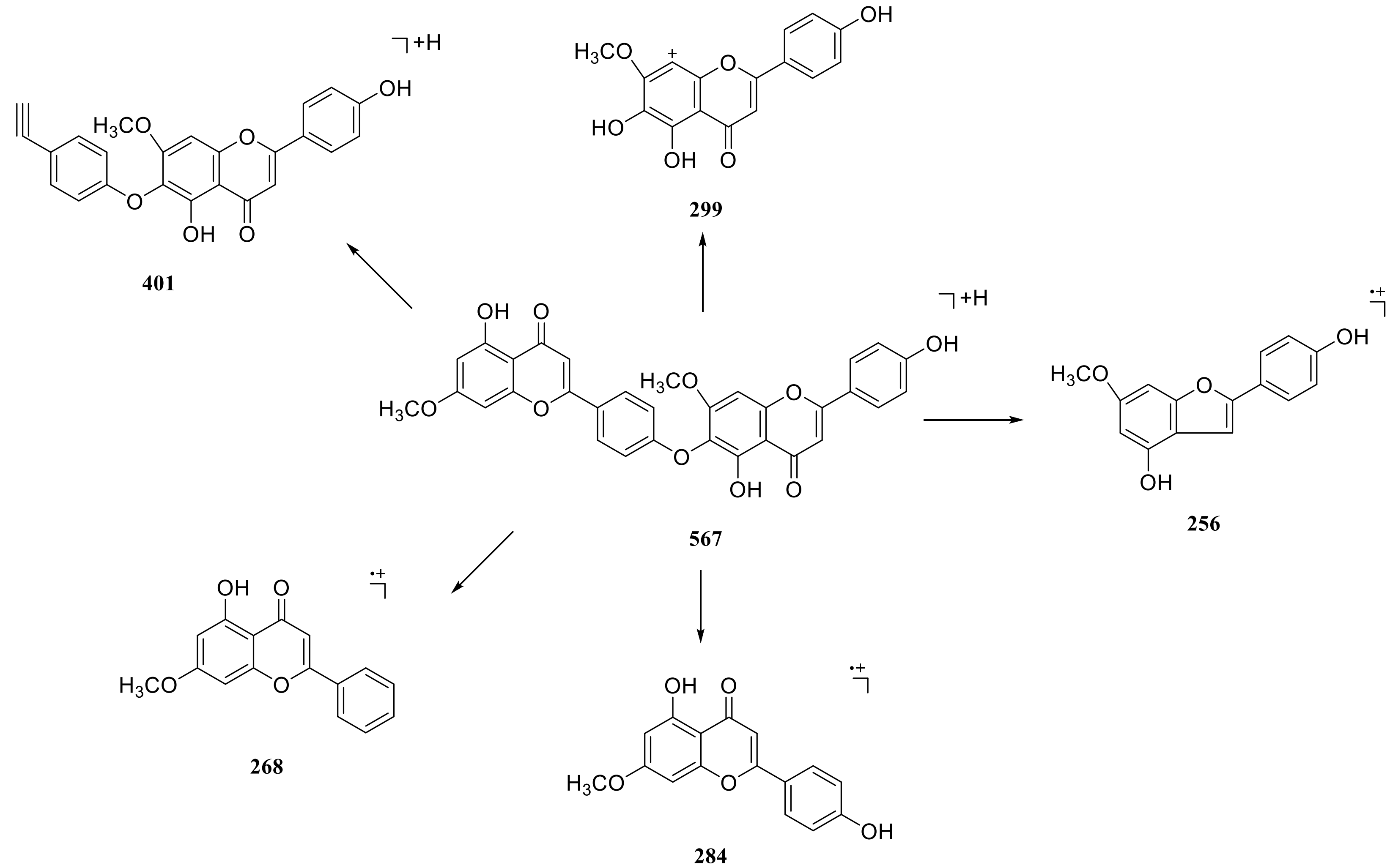

● Peak 48 (atractylenolide VI)

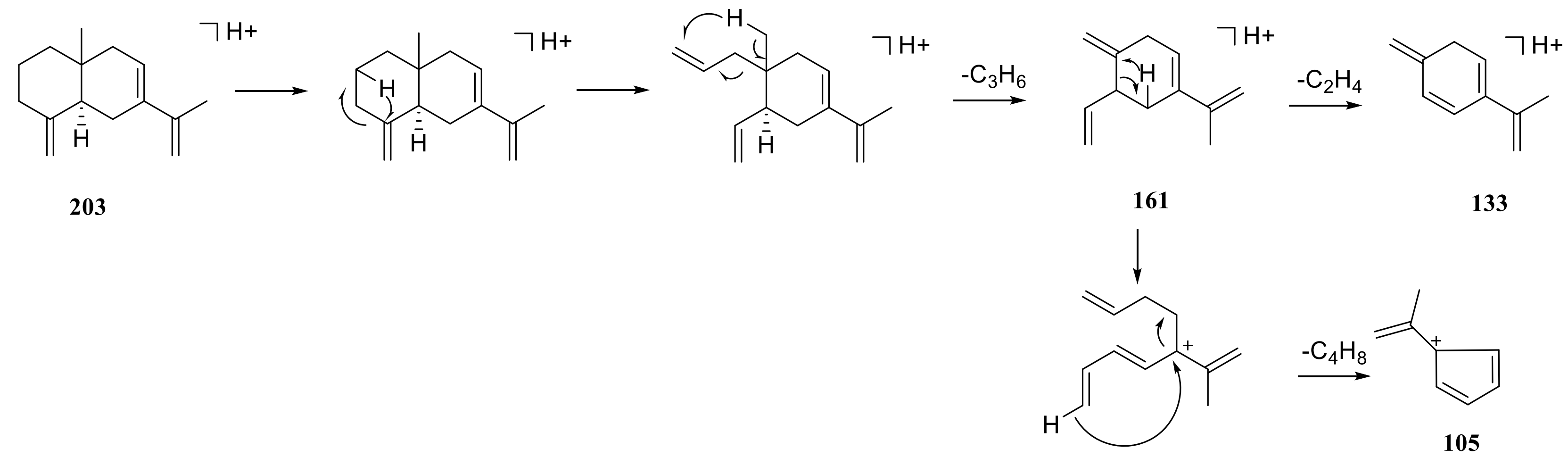
H+
H+
H+
-C3H6
H+
-C2H4
H+
203
161
133
-C4H8
105
